# Type V collagen from macrophages regulates initial collagen assembly and alignment in post-infarcted hearts

**DOI:** 10.1038/s41536-025-00430-1

**Published:** 2025-09-29

**Authors:** Xin Sun, Sarah Sigal, Maria-Alexa Cosma, Carla de Villiers, Michael Weinberger, Paul R. Riley

**Affiliations:** 1 Institute of Developmental and Regenerative Medicine, University of Oxford, Oxford, UK; 2 Department of Physiology, Anatomy and Genetics, University of Oxford, Oxford, UK; 3 Department of Cardiology, Medical University of Graz, Graz, Austria; 4 HEOR, 7 Avonbury Business Park, Howes Lane, Bicester, UK

**Keywords:** Mechanisms of disease, Myocardial infarction

## Abstract

Previous work has revealed that macrophages directly contribute collagen to the fibrotic scar in the injured hearts of zebrafish and mice. However, the functional impact of this contribution has not been investigated. Here, we characterised the deposition and ultrastructure of collagen fibrils in the forming scar of neonatal regenerative post-natal day (P)1 hearts and fibrotic P7 and adult mouse hearts after myocardial infarction (MI). Collagen type V (Col V) was the earliest deposited fibrillar collagen, coincident with macrophage recruitment to the site of injury and prior to cardiac myo-fibroblast activation. Deletion of COL5A1 in CD68+ macrophages resulted in disarrayed collagen fibrils within the nascent scar that was associated with a trend toward chamber dilation, wall thinning and compromised cardiac function. Our findings shed light on a role for macrophage-deposited Col V in establishing collagen deposition, alignment and scar stability prior to myofibroblast activation in the immediate acute phase post-MI.

## Introduction

In mice, the regeneration capability of hearts is restricted to the first week after birth. While P1 hearts can regenerate the myocardium after apical resection or infarction, P7 and adult hearts fail to regenerate. Instead, a collagen-rich scar forms to replace the necrotic myocardium to maintain the shape and function of the heart^1,2^. However, due to the scar being non-contractile, the heart undergoes pathological remodelling to compensate for the loss of function, eventually leading to heart failure.

Macrophages are classically defined as immune cells that engulf pathogens and dead cells; however, they are now known to have significantly expanded and wide-ranging roles beyond phagocytosis and efferocytosis^3^. After myocardial infarction (MI), monocytes and macrophages infiltrate the injured heart, regulating multiple pathways through all the phases of wound healing, such as necrotic tissue clearance, matrix turnover, fibroblast activation and the stimulation of angiogenesis^4–8^. Macrophages are required for heart regeneration of neonatal mice and zebrafish^9,10^. While embryonic-derived resident cardiac macrophages prohibit inflammation and promote regeneration, monocyte-derived (infiltrating) macrophages enhance inflammation and promote fibrotic repair^11,12^. A recent study also suggests that macrophages in the neonatal heart can promote regeneration not only through reducing inflammation, but also via the stimulation of cardiomyocyte proliferation^13^. Cell profiling from next-generation sequencing indicates that macrophages in regenerative hearts comprise different transcriptional subtypes compared to infiltrating populations in fibrotic hearts^5,13–16^ and that manipulating the state of macrophages may promote or impair heart regeneration^15^.

During fibrotic repair, macrophages are thought to canonically regulate ECM deposition through activation of myofibroblasts^4,7,17^. We have previously revealed an up-regulation of several ECM genes in macrophages after MI and via adoptive transfer experiments demonstrated a direct contribution of type 1 collagen (Col I) from macrophages to the forming scar in both zebrafish and mouse models^14^. This finding challenges the previous dogma that scarring in the heart arises exclusively via activated fibroblasts and highlights a potential new direct role for macrophages in the process of scar formation. However, the functional relevance of macrophage-derived collagen during cardiac fibrosis remains unknown.

Type V collagen (Col V) is classified as a regulatory fibril-forming collagen^18,19^. In humans, mutations in the *Col5a1* or *Col5a2* genes cause Ehlers-Danlos syndrome affecting the connective tissue and musculoskeletal system^20^. Col V co-assembles with Collagen I (Col I) to form heterogenous fibrils^21^. Although Col V is a minor component of the fibrotic scar relative to Collagen I and Collagen III (Col III), it has important roles in collagen fibril assembly^22^ and is essential for embryo development^23^. Following MI, Col V is a key regulator for scar size and stability and a combination of lineage tracing and RNA-FISH experiments demonstrated that the majority of Col V is synthesized by fibroblasts. Deletion of *Col5a1* from the fibroblast lineage results in abnormal collagen fibril assembly, dilation of chambers and enlarged scar size^24^.

Here, we initially examined the deposition and alignment of major fibril collagen types in regenerative (P1) and fibrotic (P7 and adult) mouse hearts post-MI. We observed altered collagen fibrillar arrangements at an ultrastructure level in regenerative P1 versus fibrotic P7 and adult hearts, consistent with transition from a more transient, unstable scar to a more permanent stable scar across the regenerative window into adulthood. We identified Col V as the earliest fibrillar collagen type deposited in response to injury and observed close association of Col V and macrophages in the infarcted area as early as 1-day post-MI, in both regenerative and fibrotic models. We hypothesized that macrophages deposit Col V at the outset post-MI to initiate fibril assembly and scar formation, prior to subsequent fibroblast activation, further collagen deposition and stabilisation of the scar. To test this hypothesis, we deleted *Col5a1* (which encodes type V collagen) in macrophages before MI surgery. Loss of *Col5a1* in macrophages (Col5a1mKO) resulted in severely dilated ventricle chambers and enlarged scar areas in infarcted mutant hearts. In the scar of Col5a1mKO hearts, the collagen fibrils were misaligned and showed evidence of significant disarray. This phenotype persisted and was not rescued by subsequent fibroblast collagen deposition. We demonstrate that macrophage-Col V is a critical component for proper collagen assembly and scar initiation and formation in the heart immediately after ischemic insult.

## Results

### Collagen deposition in regenerative and fibrotic hearts post-MI

By 21-days post-MI, adult mouse hearts form fibrotic scars to maintain structural integrity and function, as highlighted with fluorescent-conjugated wheat germ agglutinin (WGA)^25^(Supplementary Fig. 1a) and an absence of capillaries and coronary vasculature, as shown with immunofluorescent staining of endomucin^26^ (Supplementary Fig. 1b). Immunofluorescent staining confirmed the major types of fibrillar collagen, including Col I, Col III and Col V^24,27,28^ in the fibrotic scar, basement membrane, and extracellular space between cardiomyocytes (Supplementary Fig. 1c, 1c’, 1d, 1d’, 1e, 1e’). Lysyl oxidase (LOX), which catalyses collagen crosslinking^29^, was also detected (Supplementary Fig. 1f, 1f’) indicative of ECM turnover and deposition. In addition, laminin was detected within the scar, as the major component of the basement membrane^30^, indicative of the complexity of scar composition (Supplementary Fig. 1g, 1g’).

To gain insight into the ultrastructure of the fibrotic scar, we examined the scar region of the adult mouse heart at days 4, 7 and 21 post-MI (Supplementary Fig. 2) using transmission electron microscopy (TEM). The cardiac tissue near the suture was dissected and trimmed to a size of 1mm^2^ to ensure the observation plane is parallel with the short axis of the heart. In the adult heart 4-days post injury (4dpi), cardiomyocytes with fragmented sarcomeres and disarrayed mitochondria were evident, suggesting ongoing cell death (Supplementary Fig. 2a, 2d). Small clusters of collagen fibrils were observed in the low electron density area (Supplementary Fig. 2b, c, e, f). By 7-days post injury (7dpi), dying cardiomyocytes were in close association with collagen fibrils (Supplementary Fig. 2g–i). Cells of various morphologies and dense collagen fibrils were observed in the cardiomyocyte-sparse area (Supplementary Fig. 2j–l). In the adult heart 21-days post injury (21dpi), long collagen fibrils were aligned in parallel bundles within the scar (Supplementary Fig. 2m) and fibroblasts were observed with clearly visible rough endoplasmic reticulum (ER), suggesting active ECM protein synthesis and deposition (Supplementary Fig. 2n, o).

**Fig. 1:**
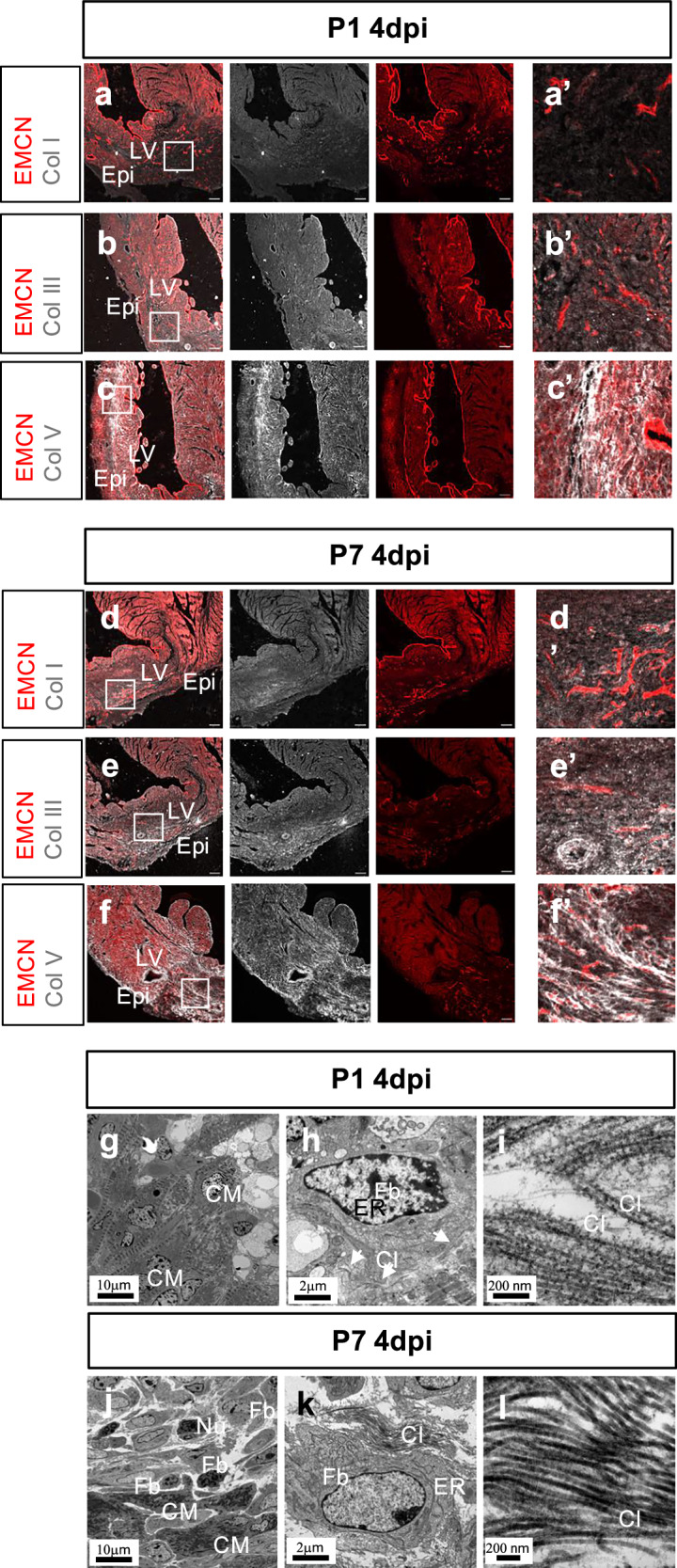
Fibrillar collagen deposition in regenerative and fibrotic mouse hearts at 4-days post-injury. Confocal imaging of an immunofluorescent-stained cross-section from P1 or P7 hearts at day 4 post-MI (4dpi) showing endomucin (EMCN)-stained vessels (red) and Collagen I (Col I, white; a, a’ and d, d’). Collagen III (Col III, white; b, b’ and e, e’) and Collagen V (Col V, white; c, c’ and f, f’). a’-f’ are magnified views of the inset boxes in (**a–f**). LV: left ventricle. Epi: epicardium. Representative images of *n* = 3, scale bar: 100 μm. Transmission electron microscopy (TEM) of the scar region of a P1 heart at 4dpi showing the injured area and cardiomyocytes (**g**). A fibroblast and surrounding collagen fibrils (white arrows) in the same P1 heart at 4dpi (**h**). Magnified collagen fibrils (**i**). Scar area of a P7 heart 4dpi showing dying cardiomyocytes, fibroblasts and collagen (**j**). A fibroblast and collagen fibrils (**k**). Magnified collagen fibrils in the P7 heart 4dpi (**l**). Scale bar as indicated. M: cardiomyocyte. Fb: fibroblast; Nu: neutrophil; ER: endoplasmic reticulum. Cl: collagen.

We next investigated the deposition of the major types of fibrillar collagen across the regenerative window at P1 and P7^31^. Hearts were harvested at days 4, 7 and 21 after surgery. The infarcted regions were confirmed by the absence of a capillary network, as revealed by immunofluorescent staining for endomucin (Fig. 1a–f, red panels).

In the P1 hearts at 4dpi, Col I was absent from the injured region (Fig. 1a, a’). Col III co-localised with endomucin-positive vessels and the endocardium but was weakly expressed in the infarcted zone (Figs. 1b, b’). In contrast, Col V was clearly detected through the infarct zone, with low abundance in the remote myocardium (Fig. 1c, c’). In the P7 hearts at 4dpi, Col I and Col III puncta were observed clustered in the infarct zone, indicating progressive collagen deposition (Fig. 1d, d, e, e’). As in the P1 4dpi hearts, Col V was enriched in the infarct region organized into a reticular network of ensuing scar (Fig. 1f, f’). These observations indicate that deposition of Col V is earlier than Col I and Col III in the scar region as an early responsive fibril-forming collagen after MI in both regenerative and fibrotic settings. TEM of injured neonatal hearts revealed low-electron density, vacuole-like structures in the regenerative P1 heart at 4dpi. Cardiomyocytes in this area were oval-shaped, with intact sarcomeres (Fig. 1g). In the scar area of the P7 heart at 4dpi, irregular shaped cardiomyocytes were observed with fragmented sarcomeres and mitochondria, and nuclei with multiple lobes, suggesting ongoing apoptosis. There were blank, low electron density gaps among the cells indicating a lack of tissue integrity (Fig. 1j). In both P1 and P7 4dpi hearts, collagen fibrils were clearly visible proximal to fibroblasts, although these were sparser in P1 versus P7 hearts (Fig. 1h, k). At higher magnification collagen fibrils in both P1 and P7 hearts were aligned, albeit those at P1 were less dense and more loosely packed, with puncta and thin filaments compared to a darker and more tight clustering of fibrils at P7 (Fig. 1i, l).

**Fig. 2:**
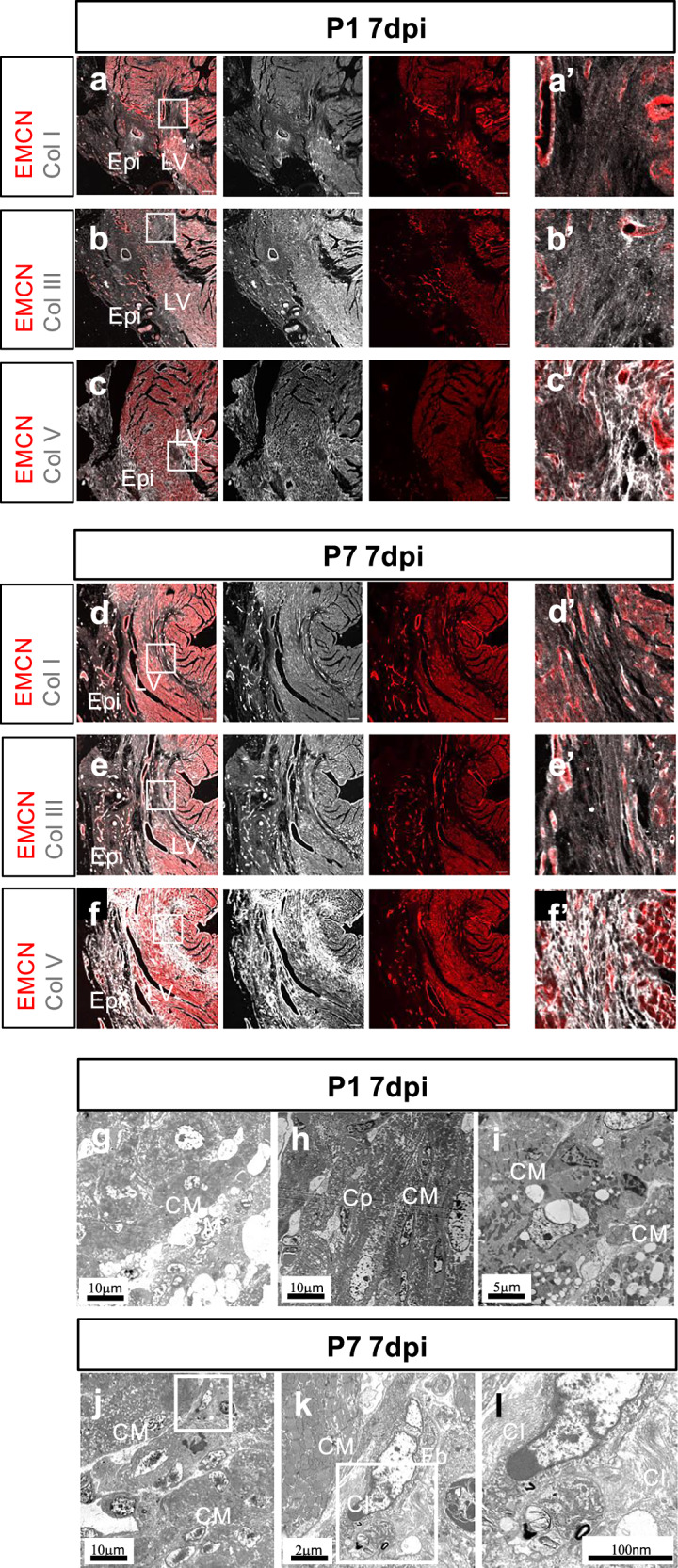
Fibrillar collagen deposition in regenerative and fibrotic mouse hearts at 7 days post-injury. Confocal imaging of an immunofluorescent-stained cross-section from P1 or P7 hearts at day 7 post-MI (7dpi) showing endomucin (EMCN)-stained vessels (red) and Collagen I (Col I, white; a, a’, d, d’:). Collagen III (Col III, white; b, b’, e, e’) and Collagen V (Col V, white; c, c’, f, f’). a’-f’ are magnified views of the inset boxes in (**a–f**). LV: left ventricle. Epi: epicardium. Representative images of *n* = 3, scale bar: 100 μm. TEM of three different area in the scar region of a P1 heart at 7dpi showing the low electron density vacuoles and cardiomyocytes (**g–i**). TEM of a section of the scar area of a P7 heart at 7dpi showing damaged myocardium and dying cardiomyocytes (**j**). A magnified view from the inset box in (**j**) showing a fibroblast and surrounding collagen fibrils (**k**). A magnified view from the inset box in (**k**) showing collagen fibril bundles (**l**). Scale bar as indicated. CM: cardiomyocyte. Cp: capillary. Fb: fibroblast. Cl: collagen.

By 7dpi, Immunofluorescent staining of the infarcted region detected accumulation of Col I and Col III in puncta patterns in P1 hearts (Fig. 2a, a’, b, b’) as compared to more fibrillar in P7 hearts (Fig. 2d, d’, e, e’). In both P1 and P7 hearts 7dpi, immunofluorescent staining detected continuous and strong signals for Col V in the endomucin-absent region (Fig. 2c, c’, f, f ’). TEM at 7dpi revealed similar clusters of low electron density, vacuole-like structures in the area proximal to the suture as at 4dpi across both P1 and P7 stages (Fig. 2g). We also observed co-existence of oval-shaped cardiomyocytes with intact mitochondria alongside cardiomyocytes showing signs of apoptosis, such as fragmented sarcomeres and mitochondria (Fig. 2g–i). At P7 7dpi, cardiomyocytes undergoing apoptosis were abundant within the ischaemic myocardium in the infarct zone (Fig. 2j). Similar to 4dpi, collagen fibrils were observed in long parallel bundles proximal to both cardiomyocytes and fibroblasts (Fig. 2k, l).

**Fig. 3:**
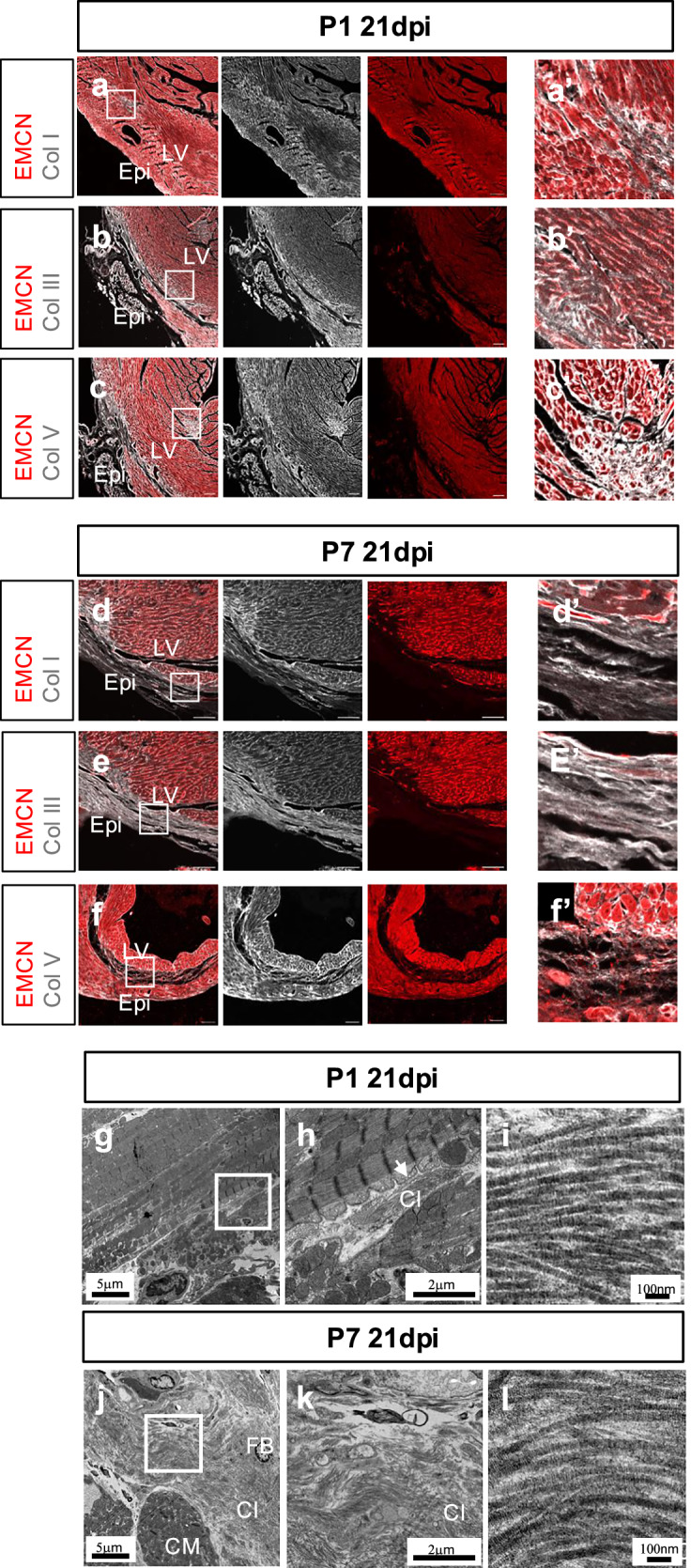
Fibrillar collagen deposition in regenerative and fibrotic mouse hearts at 21 days post-injury. Confocal imaging of an immunofluorescent-stained cross-section from P1 or P7 hearts at day 21 post-MI (21dpi) showing endomucin (EMCN)-stained vessels (red) and Collagen I (Col I, white; a, a’, d, d’:). Collagen III (Col III, white; b, b’, e, e’) and Collagen V (Col V, white; c, c’, f, f’). a’-f’ are magnified views of the inset boxes in (**a–f**). LV: left ventricle. Epi: epicardium. Representative images of *n* = 3, scale bar: 100 μm. TEM of myocardium proximal to suture of a P1 heart at 21dpi showing cardiomyocytes and collagen fibrils (**g**). Magnified view of the inset box in (**g**) showing the cardiomyocyte and the collagen fibrils (**h**). Magnified view of collagen fibrils in the same section (**i**). TEM of the scar area of a P7 heart at 21dpi showing cardiomyocytes, fibroblasts and collagen fibrils (**j**). Magnified view from the inset box of (**j**) showing abundant collagen fibrils (**k**). Magnified view of collagen fibrils in the same section (**l**). Scale bar as indicated. CM: cardiomyocyte. Cl: collagen. FB: fibroblast.

By 21-days after injury, most of the scar in the P1 hearts had been replaced with regenerated myocardium. The remaining scar was demarcated by the absence of endomucin (Fig. 3a–c, red panels) with deposition of Col I and Col III persisting in regions more distal to the original site of injury (Fig. 3a, b). Higher magnification revealed positive puncta for Col I and Col III as opposed to fibrillar staining (Fig. 3a’ b’). Col V remained enriched, proximal to the site of injury in a dense puncta pattern (Fig. 3c, c’). In the P7 hearts at 21dpi the fibrotic scar was marked by a continuous lack of endomucin staining where Col I and Col III were detected as long, parallel fibrils (Fig. 3d, d’, e, e’). Similar to Col I and Col III, Col V remained enriched in the scar and proximal myocardium (Figs. 3f, f’). TEM at 21dpi, revealed no presence of low electron density vacuoles within the regenerating myocardium and no evidence of dying cardiomyocytes. Small clusters of collagen fibrils were still evident between cardiomyocytes, consistent with the immunofluorescence results (Fig. 3g, h compare with Fig. 2a–c). Cardiomyocytes neighbouring the collagen fibrils had continuous regular sarcomeres along with orderly aligned mitochondria, indicating their viability and functionality. In the P7 21dpi heart abundant collagen fibrils were observed in the scar area (Fig. 3j, k), consistent with the patterns of Col I, Col III, and Col V immunofluorescence staining (Fig. 3d–f compare with Fig. 2d–f). Cardiomyocytes with discontinuous sarcomeres and fibroblasts were seen among collagen bundles (Fig. 3j, k). At higher magnification alignment of collagen fibrils in the P1 and the P7 hearts were morphologically similar, suggesting where residual scar was still evident at P1 it was equivalent in fibrillar maturation and stabilization to P7 (Fig. 3i, l).

### Col V is expressed early in the infarcted area coincident with the acute innate immune response

**Fig. 4:**
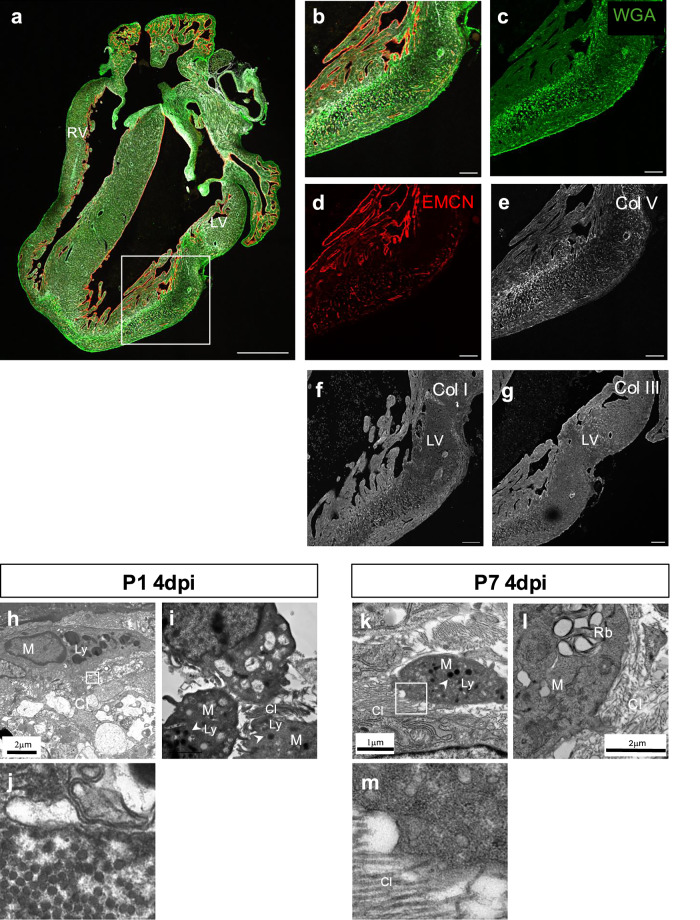
Col V is expressed in early infarcted myocardium. Confocal imaging of an immunofluorescent-stained 4-chamber cross-section from a P1 heart at 1dpi showing injured area (green), endomucin (red) and Col V deposition (white; **a–e**). Immunofluorescent staining of Col I (**f**) and Col III (**g**) of serial sections in the same region as in (**b**). LV: left ventricle. RV: right ventricle. Scale bar: 100 μm. TEM showing cells with macrophages features in a P1 heart at 4dpi and proximal collagen fibrils (**h**, **i**). A magnified view of the inset box in (**h**) showing transverse section of collagen fibrils (**j**). TEM of a P7 heart at 4 dpi showing cells with macrophage features proximal to collagen fibrils (**k**, **l**). Magnified view from (**k**) showing close association of collagen fibrils and macrophages (**m**). Scale bar as indicated. M: macrophage. Cl: collagen. Ly: lysosome. Rb: rudiment body.

Macrophages and activated fibroblasts have important roles in the repair process post-MI with activated fibroblasts the major contributors to the scar ECM^17,32^. Immunostaining with Periostin, a marker of activated fibroblasts^33,34^ in P1 and P7 infarcted hearts confirmed fibroblast activation from 4dpi at both stages (Supplementary Fig. 3). Since we detected Col V deposition by 4dpi and before Col I and Col III in both regenerative and fibrotic hearts after MI, we further explored the earliest time point of Col V deposition. In the 1dpi heart, fluorescent WGA highlighted the ECM-rich cardiac valves and the infarct area, from near the suture to the apex of the left ventricle (Fig. 4a–c). At this initial stage endomucin-expressing capillaries were already absent from the infarct region (Fig. 4d). Immunofluorescence detected Col V in the infarct zone (Fig. 4e) at this earliest time point whereas Col I and Col III were not detected in serial sections (Fig. 4f, g).

**Fig. 5:**
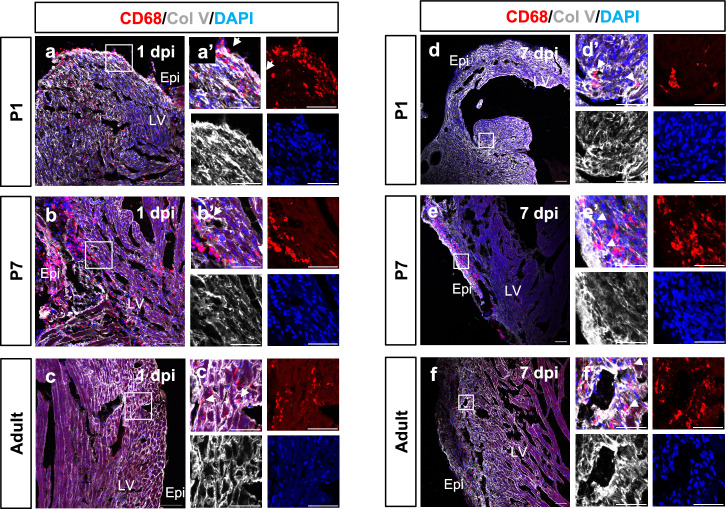
Colocalization of macrophages and Col V after injury. Confocal imaging of Immunofluorescent-stained cross-sections from P1 (**a**), P7 (**b**) and adult (**c**) hearts at 1dpi respectively, showing localization of CD68+ macrophages (red), Col V (white) and nuclei (DAPI, blue). Epi: epicardium. LV: left ventricle. Confocal imaging of immunofluorescent-stained cross-sections of P1 (**d**), P7 (**e**), and adult (**f**) hearts at 7dpi showing localization of CD68+ macrophages (red), Col V (white) and nuclei (DAPI, blue). Epi: epicardium. LV: left ventricle. Scale bar: 100 μm.

We hypothesized that the rapid deposition of Col V in the infarct zone was from macrophages, since macrophages can deposit collagen in the forming scar in both mouse and zebrafish models^14^. We initially examined P1 and P7 hearts at 4dpi with TEM and observed cells with multiple pseudopodia, containing high electron density vesicles with membrane structures recognized as lysosomes, as a marker of phagocytes (Fig. 4h, i, k) or heterogenous structures recognized as residual bodies (Fig. 4l). Thus, these cells morphologically identified as macrophages and were observed in close contact with collagen fibrils (Fig. 4j, m). In mouse hearts, macrophages are recruited to the infarct area as early as 1dpi and increase between days 2–4 dpi, returning to baseline by 7 dpi^14^. Thus, we further examined the co-localization of macrophages and Col V in regenerative and non-regenerative mouse hearts at 1dpi and 7dpi. In the P1 heart at 1dpi, clusters of CD68^+^ macrophages were detected in the epicardium and myocardium of the infarct zone. CD68^+^ cells and Col V were tightly associated (Fig. 5a, a’, white arrows). In the P7 heart at 1dpi, CD68^+^ cells were also detected in the epicardium and the myocardium, some of which outlined with Col V (Fig. 5b, b’, white arrows). In the adult heart 1dpi, Col V was also enriched in the infarct zone, in close contact with CD68^+^ cells (Fig. 5c c’, white arrows). We also examined Periostin expression in the infarct region at 1dpi. Periostin was only detected in the epicardium and sporadic cells in P1, P7 and adult hearts (Supplementary Fig 4). In the P1 heart at 7dpi, the

density of CD68^+^ positive cells in the infarct zone decreased, consistent with the previous report^14^. Small clusters of CD68^+^ cells were still detected in the dilated left ventricle (Fig. 5d) and were tightly associated with Col V (Fig. 5d’, white arrows). In the P7 heart at 7dpi, clusters of CD68^+^ cells remained in the epicardium and proximal myocardium (Fig. 5e) tightly co-localised with deposited Col V (Fig. 5e’, white arrows). In the adult heart at 7dpi, small clusters of CD68^+^ cells were observed in the myocardium associated with Col V puncta and small fibrils (Figs. 5f, f’, white arrows). Collectively this data revealed tight association of CD68+ macrophages with Col V within the infarct zone initiating as early as 1 dpi and persisting through to 7 dpi across neonatal and into adult stages. In support of macrophage expression of Col V as determined by immunostaining, we analysed a previously published single cell RNA-seq dataset^12^ which confirmed *Col5a1* (encoding COL5A1 as one of the two major isoforms contributing to Col V) was upregulated in macrophages post-MI (Supplementary Fig. 5).

### Loss of *Col5a1* in macrophages results in increased scar size, pathological remodelling and abnormally assembled collagen fibrils

**Fig. 6:**
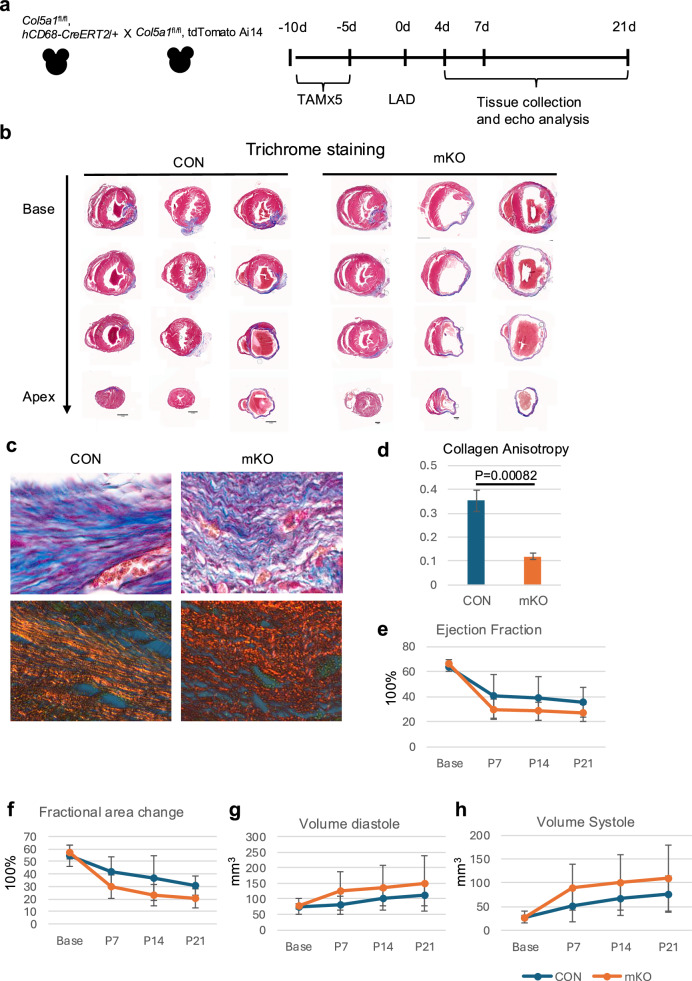
Col V deletion from macrophages results in altered scarring and compromised collagen alignment. Schematic showing the Col5a1 deletion strategy in CD68^+^ macrophage lineages. *Col5a1*^*fl/fl*^, *hCD68-CreERT2/*^*+*^ males were crossed with *Col5a1*^*fl/fl*^, *tdTomatoAi14/tdTomatoAi14* females. Progenies were administered 200 mg tamoxifen for 5 days and then subjected to LAD ligation surgery and analysed at the following timepoints (**a**). Masson-Trichrome staining of serial sections from three controls and three mKO samples showing the dilated and thinned left ventricles (**b**). Scale bars: 5 mm. Magnified Masson-Trichrome staining of representative sections from a control (CON) and an mKO heart (**c**, upper panel). Polarized laser microscopy on picrosirius red staining (lower panel) of serial sections from the same control and mKO hearts showing the collagen fibrils (red) in the left ventricle myocardial wall (**c**, lower panel). Scale bars 10 μm. Degree of anisotropy analysed with Fibriltool based on polarized laser microscopy on picrosirius red staining of serial sections from the control (CON) hearts and mKO hearts (**d**) *N* = 3 per group; *p* = 0.0082. Ejection fraction (EF, **e**), Fractional area change (FAC, **f**) and end diastolic (**g**) and systolic volumes (**h**) as determined by echocardiography (ECHO) comparing controls and Col5a1mKO mice at baseline nad different time points post-MI. *N* = 4 per group; EF, baseline p = 0.78, 7dpi *p* = 0.32, 14dpi *p* = 0.31, 21 dpi *p* = 0.24; FAC, baseline *p* = 0.60, 7dpi *p* = 0.51, 14dpi *p* = 0.22, 21 dpi =0.09; EDV, baseline *p* = 0.94 7dpi *p* = 0.23, 14dpi *p* = 0.41, 21 dpi =0.47; ESV, baseline *p* = 0.96, 7dpi *p* = 0.24, 14dpi *p* = 0.34, 21 dpi =0.19.

Deletion of *Col5a1* in cardiac fibroblasts increased fibrosis and scar size after MI^24^. Given the immediate early expression of Col V post-MI, prior to fibroblast activation and as associated with macrophages in the infarct region, we crossed *hCD68-CreERT2* mice with *Col5a1*floxed mice to specifically delete *Col5a1* from tissue-resident macrophages^23,35^. The human CD68 promoter drives inducible Cre recombinase expression in tissue resident macrophages and Ly6C^low^ monocytes in the spleen but not recruited inflammatory monocytes^35^. Tamoxifen was administered to hCD68CreERT2, *Col5a1*^flox/flox^, *Rosa26-tdTomatoAi14*^36^ animals and their littermates that lacked the Cre transgene for 5 days, followed by LAD ligation and harvesting of hearts for analysis over a time course (Fig. 6a).

We first examined the expression of the reporter tdTomato to evaluate the Cre recombination efficiency. At 4dpi, cells expressing tdTomato were visible in the *CD68-CreERT2* + , *Col5a1*^flox/flox^ (Col5a1mKO) hearts (Supplementary Fig. 6a–h) but not those without Cre (CON) (Supplementary Fig. 6i–l). CD68 and Lyve1, both macrophage markers^37–39^, were detected in the infarct region and remote region. TdTomato-positive cells overlapped with CD68 and partially with Lyve1 (Supplementary Fig. 6a–d, 6e–h) consistent with a tissue-resident macrophage source. Of note, the majority of CD68^+^ macrophages enriched in the wound area were not tdTomato positive nor Lyve1 positive, consistent with the known heterogeneity of monocyte and macrophage populations within infarcted hearts^40^. At this stage, loss of Col V associated with CD68+ cells was evident (compare Supplementary Fig. 7a-c and 7d-f). By 7dpi, a larger number of tdTomato^+^/CD68^+^/Lyve-1^+^ cells were observed in the infarct region (Supplementary Fig. 8a-d). No tdTomato^+^ cells were present in the remote myocardium (Supplementary Fig. 8e-h) and none were observed in littermate Cre-negative control hearts. (Supplementary Fig. 8i-l). Loss of Col V did not affect recruitment of CD68+ cells, as the density of CD68+ cells in the infarcted regions was comparable at 7dpi (compare Supplementary Fig. 9a-c and 9d-f).

Histological analysis of the Col5a1mKO hearts at 21dpi revealed significantly dilated chambers and thinned ventricle walls compared with littermate controls (Fig. 6b). Masson trichrome staining detected thick, parallel collagen fibril bundles in the mature scar of the control hearts, whereas in the Col5a1mKO hearts, collagen fibrils were wavy and thin (Fig. 6c, top panel). Picrosirius red staining and polarized light microscopy of serial sections detected red and yellow-stained collagen fibrils formed into long, parallel bundles in the control hearts, while collagen fibrils in Col5a1mKO hearts were fragmented and misaligned (Fig. 6c, lower panel). We measured the degree of anisotropy of collagen using *Fibriltool*^41^. Analysis of polarized light microscopy images of picrosirius red stained serial sections showed decreased anisotropy in Col5a1mKO compared with the control hearts (CON 0.35±0.04, mKO 0.12±0.01, *n* = 3 animals per each genotype, *p* = 0.0082), indicating a higher degree of misalignment of collagen fibres in the Col5a1mKO hearts (Fig. 6d).

To evaluate the cardiac function of Col5a1mKO mice, we performed echocardiography on the animals at different time points after LAD surgery. We observed consistently reduced contractile performance (ejection fraction, fractional area change) in the Col5a1mKO mice from 7 days after injury which persisted to 21 days (Fig. 6e, f. Ejection fraction percentage: base: CON 63.8±3.7 vs mKO 66.2±3.7, *p* = 0.78; 7dpi: CON 40.3±17.7 vs mKO 29.8±8.0, *p* = 0.32; 14dpi: CON 38.7±17.4 vs 28.4±7.2, *p* = 0.31; 21dpi: 35.4±11.8 vs 26.6±6.8, *p* = 0.24. Fractional area change percentage: base: CON 54.7±8.6 vs mKO 57.4±5, *p* = 0.60; 7dpi: CON 42.1±11.6 vs mKO 29.8±9.6, *p* = 0.51; 14dpi: CON 36.8±17.7 vs mKO 23.5±8.6, *p* = 0.22; 21dpi: CON 30.9±7.5 vs mKO 20.3±7.6, *p* = 0.09; *n* = 4 animals per genotype). Col5a1mKO animals also revealed elevated pathological remodelling, with increased end diastolic and systolic volumes from 7dpi to 21dpi (Fig. 6g, h. Volume diastole mm^3^: base: CON 75.7±9.0 vs mKO 76.6±25.2, *p* = 0.94; 7dpi: CON 79.8±29.9 vs mKO 125.8±62.6, *p* = 0.23; 14dpi: CON 102.9±25.4 vs mKO 136.2±71.8, p = 0.41; 21dpi: CON112.2±34.2 vs mKO 148.8±90.0, *p* = 0.47. *n* = 4. Volume systole mm^3^: base: CON 27.3±3.9 vs mKO 27.1±12.6, *p* = 0.96; 7dpi: CON 51.6±34.0 vs mKO 90.4±49.0, *p* = 0.24; 14dpi: CON 66.2±34.5 vs mKO 100.5±58.2, *p* = 0.34; 21dpi: CON 75.4±38.0 vs mKO 110.4±69.8, *p* = 0.19; *n* = 4 animals per genotype). These results indicate trends towards compromised cardiac function in Col5a1mKO hearts.

To investigate whether Col V from the macrophages is required for myofibroblast activation, we immunostained for the myofibroblast marker Periostin and Vimentin, a marker of epithelial-mesenchymal transition (EMT)^42^ in the hearts of Col5a1mKO and their littermate controls at both 4 and 7dpi. At 4dpi, Periostin was decreased in the Col5a1mKO infarct region compared to control hearts (Supplementary Fig. 10a–b, *n* = 3), but this difference was not evident by 7dpi (Supplementary Fig. 10c–d, *n* = 3). The expression of Vimentin was comparable between Col5a1mKO and control hearts at both time points (Supplementary Fig. 10e–h, *n* = 3). This suggests that Col V deletion from macrophages does not compromise activation of fibroblasts or EMT in the infarct region.

**Fig. 7:**
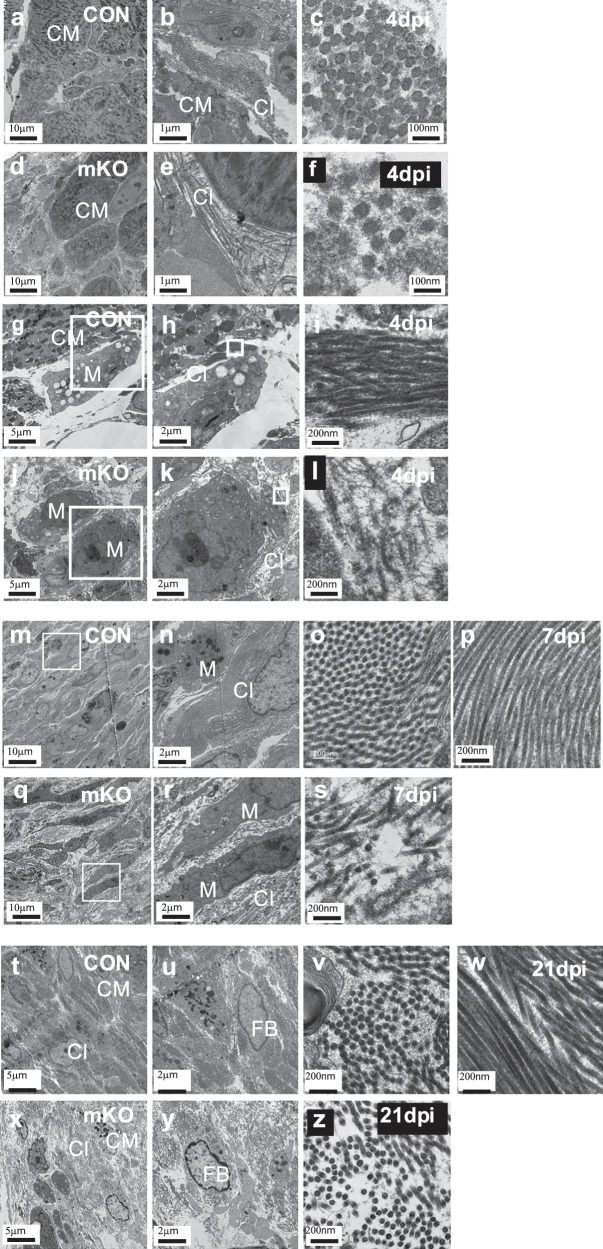
Ultrastructure defects in the extracellular matrix (ECM) of macrophage-Col V depleted mice. TEM showing the morphology of cells and associated collagen fibrils in infarcted hearts of *Col5a1*^*fl/fl*^ (CON) and *Col5a1*^*fl/fl*^*, CD68-CreERT2*^*+*^ (mKO) mice at 4dpi (**a–l**), 7dpi (M-S) and 21dpi (T-Z). Lower magnification views of serial sections of the injured area within the myocardium of a CON (a) and mKO heart at 4dpi (**d**). Collagen fibril bundles in the myocardium of CON (**b**, **c**) and mKO hearts (**e**, **f**). Macrophages in close proximity to collagen fibrils in CON (**g**) and mKO hearts (**j**) at lower magnified views highlighting differences in collagen fibril bundle formation, deposition and alignment (**h**, **i**, **k**, **l**). Lower magnification views of serial sections of the injured area within the myocardium of a CON (**m**) and mKO heart at 7dpi (**q**) with higher magnification insets (**n**) and (**r**). Higher magnification to show differences in collagen fibril bundles and fibres in the myocardium of CON (**o**, **p**) and mKO hearts (**s**). Macrophages proximal to collagen fibrils in CON (N) and mKO hearts (R). Lower magnification views of serial sections of the injured area within the myocardium of a CON (**t**) and mKO heart at 21dpi (**X**). Magnified view from (**t**) showing collagen fibrils associated with a fibroblast (**u**) and collagen fibrils in transverse section (**v**) and longitudinal sections (**w**) in CON hearts. Magnified view from (**x**) showing collagen fibrils and a fibroblast (**y**) and disorganised collagen fibrils in transverse section in the mKO heart (**z**). Scale bar: as indicated. CM: cardiomyocyte. Cl: collagen. M: macrophage. FB: fibroblast.

We next analysed the collagen fraction of the control and Col 5a1mKO hearts from Masson’s Trichrome-stained hearts at 4dpi, 7dpi (Supplementary Fig. 11a, b) and 21dpi (Fig. 6b)^43^ and observed higher collagen fraction in Col5a1mKO hearts than the control (CON 0.17±0.05 vs mKO 0.21±0.03 at 4dpi, *p* = 0.373, CON 0.13±0.04 vs mKO 0.22±0.10 at 7dpi, *p* = 0.278, CON 0.27±0.06 vs mKO 0.36±0.04 at 21dpi *p* = 0.158; *n* = 3 animals per each group, Supplementary Fig. 11c). Although the differences were not statistically significant, the trend of increased collagen fraction in Col5a1mKO hearts was consistent across the stages analysed. To further investigate the impact of Col V deletion from the macrophages on scar ultrastructure and collagen fibril arrangement after MI, we performed TEM on the infarcted myocardium. At 4dpi, in both control and Col5a1mKO, cardiomyocytes were observed undergoing cell death proximal to collagen-rich regions (Fig. 7a, d). In control myocardium, collagen fibrils were tightly packed together into thick bundles (Fig. 7b), whereas in the Col5a1mKO hearts, the collagen fibrils were disarrayed and loosely packed (Fig. 7e). Transverse EM sections of collagen fibrils revealed an irregular arrangement and structure of forming collagen fibrils in the Col5a1mKO hearts (Fig. 7c, f). Macrophages, as identified by the presence of residual bodies and evident lysosomes, were observed in the scar region (Fig. 7g, j), proximal to collagen fibrils that were either aligned and tightly packed together in controls (Fig. 7h, i), or fragmented and disarrayed in the mutants (Fig. 7k, l). The presence of misaligned and thin filaments with irregular puncta suggests that the collagen fibrils in the mutant hearts were not properly assembled or maintained in the absence of a source of macrophage-derived Col V (Fig. 7i, l).

By 7 dpi, collagen fibrils were observed together with readily identifiable macrophages, fibroblasts, and cardiomyocytes in the scar regions of both control and Col5a1mKO hearts (Fig. 7m, n, q). The collagen fibrils in the Col5a1mKO heart were loosely configured and disarrayed, resulting in low electron density areas between cells, indicating compromised tissue integrity (Fig. 7m, q). Mutant fibrils failed to form long, parallel bundles and instead were misaligned (containing orthogonal fibrils), fragmented and disorganized (Fig. 7r, s) relative to control hearts (Fig. 7o, p). Cells within the Col5a1mKO scar also showed abnormal morphology, potentially due to the altered ECM (Fig. 7m, q). By 21dpi, collagen in the scar tissue of Col5a1mKO remained less dense and fibrils were fragmented and disarrayed relative to controls (Fig. 7t, x). In both Col5amKO and control samples, fibroblasts and dying cardiomyocytes were visible among the collagen fibres (Fig. 7t, u, x, y). There were less puncta and thin filaments among the collagen fibrils in the Col5a1mKO at this later time point (Fig. 7v, w, z), potentially due to maturation or re-organization of the collagen and/or restoration of the fibril architecture through an alternative activated myofibroblast source of Col V. Collectively, these data reveal that macrophage-Col V is an essential early source of ECM post-MI. Col V from macrophages acts immediately following the initial insult to establish collagen fibrillar ultrastructure and alignment, which in turn ensures scar stability and maintains heart structure and function. Whereas other sources of Col V (most likely from activated fibroblasts) may restore the collagen fibrillar ultrastructure at later stages post-MI (by 21dpi) this cannot compensate for the essential early function of Col V from macrophages within the scar region.

## Discussion

The adult mammalian heart has limited regenerative capacity; consequently, following injury or insult the formation of a reparative fibrotic scar is essential for wound repair (to prevent ventricular rupture) and maintenance of cardiac function^32^. In neonatal hearts (both in mice and human infants), the scar is transient and is eventually replaced by newly formed myocardium^1,2,44^. One week after birth, and during the transition to adulthood in mice, repair is orchestrated by fibrosis and mature scar formation, which becomes a stable non-contractile component of the myocardium wall. The formation, composition, ultrastructure and maturation of the fibrotic scar, as determines remodelling and function of the injured hear,t has not been comprehensively investigated. An increasing number of studies highlight the importance of ECM composition and fibril alignment in the process of scar formation. For example, deletion of the cellular communication network 1(CCN1), which has antifibrotic activity, resulted in enlarged scar size and increased animal mortality due to ventricle rupture^45,46^. Changing the topological arrangement of cardiac fibroblasts altered their gene expression profile and affected cardiomyocyte morphology^47^. Collagen fibrils, once formed, have been suggested to remain static without turnover^48^, although more recently the breakdown of collagen bundles has been demonstrated in mammalian tissues, suggesting the importance of collagen homeostasis^49^. The dynamics of collagen fibrils and bundles in the infarcted heart remain largely unknown, and their turnover in the scar post-MI has not been characterized.

Here, we present the spatial-temporal dynamics of the major fibrillar collagens deposited in response to MI in both regenerative and non-regenerative mouse models. Transmission electron microscopy revealed the ultra-structure of the infarcted myocardium, including cell types associated with differences in the collagen fibrillar arrangement and scar morphology in regenerative and non-regenerative hearts. The morphology of collagen fibrils changed significantly from early to late stages post-MI, suggesting a highly dynamic assembly process. We demonstrate that Col V responds to injury during the first 24 hours after ischaemic insult, in both regenerative and fibrotic models, preceding both fibroblast activation and deposition of the more abundant type I and type III collagens. Col V was previously identified as being up-regulated from 3 days post-MI, originating from Col1a2 and TCF21 lineage-derived fibroblasts^24^. Our data indicate deposition at an earlier time point, suggesting Col V may play a role in an immediate fibrotic response to patch up the area of injury.

Col V has long been implicated in the regulation of collagen fibril assembly^21,23,50^ and loss of *Col5a1* results in embryonic lethality due to deposition of abnormal collagen fibres during development^22^. Mice heterozygous for *Col5a1* have increased fibrosis in the myocardium, recapitulating human Ehlers-Danlos syndrome^51^ and conditional targeting of *Col5a1* in fibroblasts, before LAD ligation, resulted in enlarged scar size, dilated chambers and compromised cardiac function^24^. We reveal herein that macrophage-specific deletion of *Col5a1* recapitulated the phenotypes of *Col5a1* loss in fibroblasts^24^, building on our previous findings that macrophages can deposit collagen in zebrafish and mouse heart injury models^14^. This was a surprising finding, for which a potential explanation is that the Col1a2- and Tcf21-Cre recombinase lines employed previously to target cardiac fibroblasts^24^ may have expressed Cre in macrophages. Single-cell sequencing has confirmed *Col1a2* up-regulation in macrophages in mouse hearts post-MI^14^ and expression of TCF21 has also been reported in macrophages^52^. A distinct study reported low expression of *Tcf21* in myeloid cells in infarcted hearts^53^. This latter discrepancy may reflect differences in macrophage sub-populations expressing *Tcf21*/TCF21, given the known monocyte-macrophage heterogeneity in the injured heart^40^.

Despite there being an important source of Col V from activated fibroblasts, in the context of regulating scar size post-MI^24^, this was unable to “rescue” or compensate for the early loss of Col V from macrophages, as evidenced by our longitudinal histology and ECHO studies. We observed a close association of macrophages and Col V as early as one day post-MI and our lineage-tracing experiments revealed *Col5a1* deletion was evident in a sub-population of macrophages, which was sufficient to result in prominent defects in collagen fibril assembly and impaired scar formation. Macrophages are among the earliest and most abundant innate immune cells that respond to damage-associated signals to infiltrate the area of injury and begin the clean-up and repair process^54^. They play a number of key roles post-MI^5,55–57^, including phagocytosis of dead and dying cells, removal of neutrophils, fine-tuning of inflammation^11^, stimulating angiogenesis^8^ and lymphangiogenesis^58^, ECM remodelling and turnover through matrix metalloproteinase production^59,60^ and activating fibroblasts^16,17,61^. The composition of macrophages in the post-MI heart is heterogeneous and highly dynamic and changes during the pro-inflammatory and anti-inflammatory/pro-repair phases of wound healing^4^. Our findings suggest that repair, as directed by Col V macrophages, is initiated significantly earlier than as classically described^54^ and occurs in parallel with the process of necrotic tissue removal. In the absence of Col V from macrophages, the collagen fibrils fail to assemble properly, compromising scar formation and subsequent maturation which ultimately results in adverse cardiac remodelling and impaired function.

Future work studying collagen dynamics during scar formation may offer insights on mitigating, or even reversing, cardiac fibrosis. Novel therapeutic strategies targeting ECM-relevant mechanisms have made encouraging progress in this regard^17,62,63^. Chimeric antigen receptor (CAR) T cells engineered to target cardiac fibroblasts expressing Fibroblast activating protein (FAP) have proven effective to reduce fibrosis^64,65^. Genetic deletion and pharmacological inhibition of FAP also improved cardiac function after MI^66^. An inhibitor against αvβ3 and αvβ5 integrins rescued the defects caused by *Col5a1* conditional deletion in the cardiac fibroblasts^24^. Our findings impart an essential early function on macrophage-derived collagen, as a determinant of scar stability and function; thus, targeting macrophage polarization may be a promising approach to regulate ensuing fibrosis in the setting of acute MI.

## Methods

Listings of antibodies; chemicals, peptides, and recombinant proteins; experimental models (including organisms and strains) and software and algorithms utilised in the study Table 1.

**Table 1:** Key resources

Antibodies		
Rabbit monoclonal anti-mouse collagen I	Abcam (1:200)	Cat# Ab34710
Rabbit monoclonal anti-mouse collagen III	Abcam (1:200)	Cat# Ab7778
Rat monoclonal anti-mouse EMCN (clone V.5C7)	Santa Cruz Biotech (1:50)	Cat# sc-53941
Rabbit monoclonal anti-mouse collagen V	Abcam (1:200)	Cat# Ab7046
Rabbit polyclonal anti-mouse Periostin	Abcam (1:200)	Cat# Ab14041
Rat monoclonal anti-mouse Vimentin	Abcam (1:200)	Cat# Ab92547
Rabbit polyclonal anti-mouse total laminin	Abcam (1:200)	Cat# Ab11575
Donkey anti-rat IgG Alexa Fluor 488	Thermofisher	Cat# A21208
Donkey anti-rat IgG Alexa Fluor 555	Abcam	Cat# Ab150154
Donkey anti-rabbit IgG Alexa Fluor 555	Thermofisher	Cat# A32794
Donkey anti-rabbit IgG Alexa Fluor 647	Thermofisher	Cat# A31573
**Chemicals, Peptides, and Recombinant Proteins**		
Paraformaldehyde (PFA) solution 4% in PBS	Santa Cruz Biotech	Cat# 281692
DAPI solution	Invitrogen	Cat# 62248
Wheat germ agglutinin (WGA)	ThermoFishcer	Cat# W11261
**Experimental Models: Organisms/Strains**		
Mouse: B6.Cg-Gt(ROSA)26Sor^tm14(CAG-tdTomato)Hze^/J	The Jackson Laboratory	JAX: 007914
**Software and Algorithms**		
Fiji-Image J	NIH	N/A
FV1000	Olympus	N/A

### Mouse lines

Genetically modified mouse lines were kept on a pure C57BL/6 background. *Col5a1fl/fl* mice were crossed with hCD68-CreERT2 mice to generate *Col5a1*^*fl/fl*^, hCD68-CreERT2 stud males. *Col5a1*^*fl/fl*^ were crossed with Ai14 tdTomato (Table 1) to generate *Col5a1*^*fl/fl*^, tdTomato/tdTomato females for the lineage tracing. Mice were housed and maintained in a controlled environment by the University of Oxford Biomedical Services according to a United Kingdom Home Office establishment licence (reference XEC303F12).

### Neonatal and adult mouse models of MI

MI was performed by ligation of the left anterior descending (LAD) coronary artery. For adult mice, we carried out surgery on animals of body weight above 20 grammes (usually between 8–10 weeks of age). After 5 days continuous oral gavage of tamoxifen and 5 days interval (2 mg per animal per day^35^;, the animals were subjected to LAD ligation as described previously^67^. Briefly, animals were anesthetized with 2.5% isofluorane and placed under assisted external ventilation through the insertion of an endotracheal tube. The LAD coronary artery was ligated with an 8-0 suture. MI in neonatal mice has been described in detail previously^31^. All animal procedures were carried out with local ethical approval (AWERB) at the University of Oxford and under the regulation of a United Kingdom Home Office project licence (reference PP3194787) in full compliance with the Animals (Scientific Procedures) Act 1986 (A(SP)A, revised 2012). Animals were euthanized according to the UK Home Office designated schedule 1 method of cervical dislocation.

### Fluorescent Immunohistochemistry

Tissues were harvested and dissected at the required stages in PBS and fixed with 4%PFA (Table 1) overnight. To prepare tissue sections, the fixed samples were embedded in OCT compound embedding medium. Serial sections were prepared with a cryostat microtome. Alternatively, the samples were dehydrated in an ethanol gradient and embedded in paraffin. The paraffin blocks were then sectioned into 10 um sections and mounted onto glass slides.

For cryosections, glass slides or coverslips were washed twice with PBS to remove the OCT then permeabilize with 0.5% TritonX-100 in PBS for 10 min and washed with PBS afterwards. For paraffin sections, the slides were dewaxed in two rounds of histoclear then hydrate through ethanol gradient. For antigen retrieval, the slides were incubated in 10 ug/ml proteinase K, pH 8.0, for 15–20 min at 37 °C. The proteinase K was then washed off with PBS. The slides were permeabilized with 0.5% TritonX-100.

The sections were then blocked in 10% donkey or goat serum, 1%BSA and 0.1%TritonX-100 in PBS for more than 1 h at room temperature. Next, samples were incubated with primary antibodies (Table 1) diluted in the blocking solution at the concentration indicated above overnight at 4 °C. The next day, samples were washed three times in 0.1% TritonX-100 in PBS for 10 min then incubated with corresponding fluorescent secondary antibodies (Table 1) diluted at 1:200 in 0.1% TritonX-100 in PBS for 1 h After three washes with 0.1%TritonX-100 and 10 min of DAPI (Table 1) staining, slides were mounted in 50% glycerol in PBS and imaged with Olympus FV1000 confocal microscope. Images were analysed with FIJI (Table 1).

For Masson-trichrome staining and Picrosirus red staining, paraffin sections were dewaxed and washed in PBS. Abcam Trichrome stain kit (Ab150686) and Picrosirius red stain kit (Ab150681) were used to stain the sections. The slides were then dehydrated in ethanol gradient and mounted in DPX Mounting medium (Sigma 06522). Stained slides were scanned on Zeiss Axioscan 7 or imaged with Olympus BX53 laser polarizing microscope.

### Transmission electron microscopy

Samples were fixed and stained following a previously published protocol^68,69^. After mounting, the samples were sectioned at 90 nm on Leica Ultracut 7 Ultramicrotome. The sections were observed on JEOL1400 120 kV and JEOL 2100 plus 200 kV TEM.

### Echocardiography

Left ventricular function was analysed by echocardiography at baseline 3 days prior to surgery and at 7-, 14-, and 21-days post-injury using the Vevo 3300 (Visualsonics) and a UHF57x 57–25 MHz transducer (Visualsonics).

Mice were anaesthetised in an induction chamber at 4% isoflurane before being transferred to a heated platform set to 38.5 °C and placed in a nosecone. The mice were arranged supine and maintained under anaesthesia for the duration of the imaging at 1.5% isoflurane. Oxygen flowrate was kept at a consistent 1 L/min throughout. Mouse ECG, heart rate, and breathing rate were monitored using the platform’s inbuilt functions. Mouse core temperature was monitored using a thermal rectal probe and maintained at 37 °C through the platform’s heating function and the use of an infrared warming lamp when needed. The mice were shaved using a clipper followed by hair-removal cream.

The transducer probe was stabilised by a rail-mounted holder system. For each heart, Brightness-mode (B-mode) 300-frames clips encompassing multiple contraction cycles were taken of the Parasternal Long-Axis (pLAX) view, followed by mid-ventricular, apical, and basal orthogonal parasternal short axis (pSAX) views by a genotype-blinded researcher. Post-acquisition analysis was carried by the same blinded researcher.

In the licensed software VevoLab (Visualsonics), diastolic and systolic lengths of the left ventricle were measured from the pLAX, and the endocardium of the left ventricle was traced manually at systole and diastole for each of the three pSAX B-modes. Each measurement was taken in duplicate, from distinct contraction cycles. Mice post-myocardial infarction occasionally presented with arrythmias and thus measurements were only taken from non-arrhythmic contractions.

From the average of these measurements, cardiac functional parameters (namely: cardiac output, ejection fraction, fractional area change, fractional shortening, stroke volume, end diastolic volume, and end systolic volume) were calculated by the VevoLab software using Simpson’s bi-plane method.

### Collagen quantification

Collagen fraction was deconvoluted using ImageJ plugin from Masson’s trichrome-stained serial sections. The area of blue collagen fibres was isolated by adjusting the threshold in ImageJ to cover the whole scar region. The area was then quantified with ImageJ. The red component representing the total area of the section was measured and the ratio of collagen area against the total area was calculated.

### Anisotropy analysis

The anisotropy of collagen fibres was analysed using the Fibriltool plug-in within ImageJ. Serial sections were stained with picro-sirius red and imaged with polarised light microscopy. The red and yellow-stained collagen areas were selected and analysed with Fibriltool.

### Statistical analysis

All data are presented as mean ± standard error of the man (SEM). Statistical analysis was performed on Microsoft Excel. The statistical significance between two groups was determined using an unpaired two-tailed Student’s *t*test, these included an F-test to confirm the two groups had equal variances. A value of *p* < 0.05 was considered statistically significant.

## Supplementary information

Supplementary Information

## Data Availability

No datasets were generated or analysed during the current study.
