# Supplementary material for: Type V collagen from macrophages regulates initial collagen assembly and alignment in post-infarcted hearts
Source: NPJ Regen Med. 2025 Sep 29;10:44. doi: 10.1038/s41536-025-00430-1 (PMC12480006; doi:10.1038/s41536-025-00430-1)
Supplement: Supplementary file 1 — Supplementary Information [file 41536_2025_430_MOESM1_ESM.pdf]

Supplementary Fig 1. Major collagen components in the mature adult heart scar post-MI.

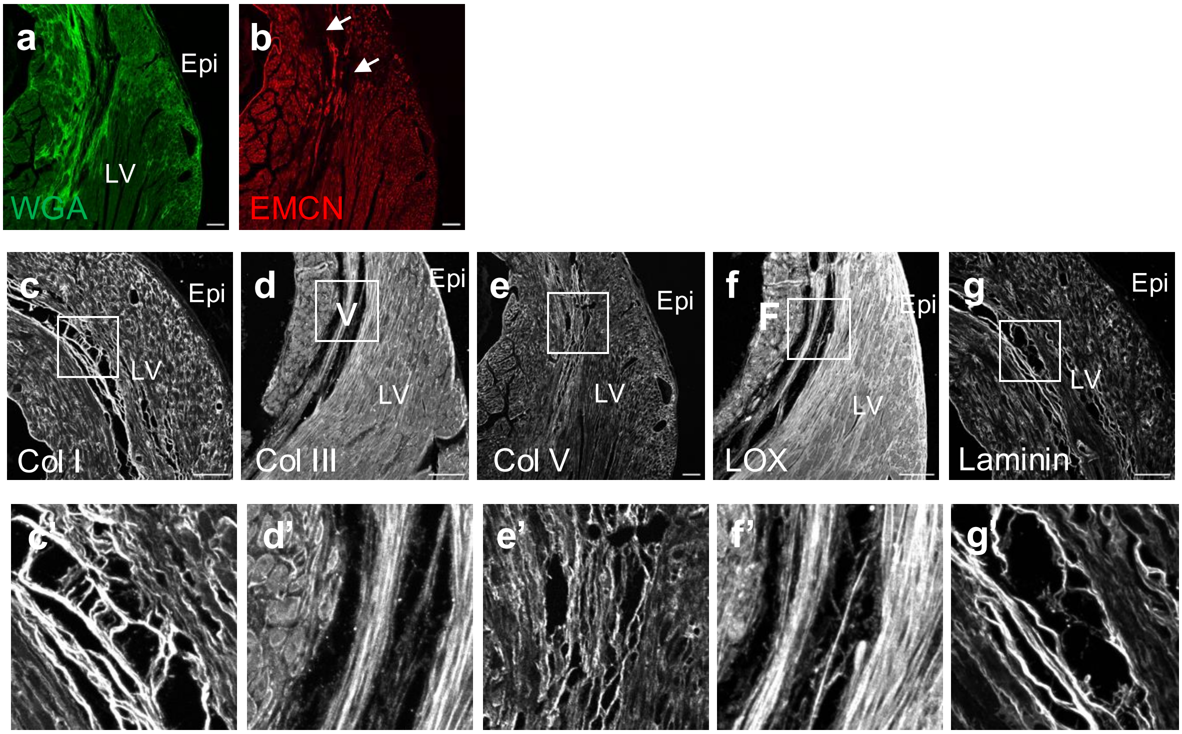

Supplementary Fig.1. Major collagen components in the mature adult heart scar post-MI.

Fluorescent conjugated wheat germline agglutinin (WGA) highlights the scar area (green) in a cross section of an adult heart at 21dpi (a). Immunofluorescent staining with endomucin (EMCN) showing absence of vessels (b) and the major collagen (c, c', d, d', e, e') and ECM components (f, f', g, g') in the scar area of a serial section from the same heart. c', d', e', f', g' are magnified view of the inset boxes of c-g. Scale bar: 50μm.

Supplementary Fig. 2. Transmission Electron microscopy of the ultrastructure of the infarct scar.

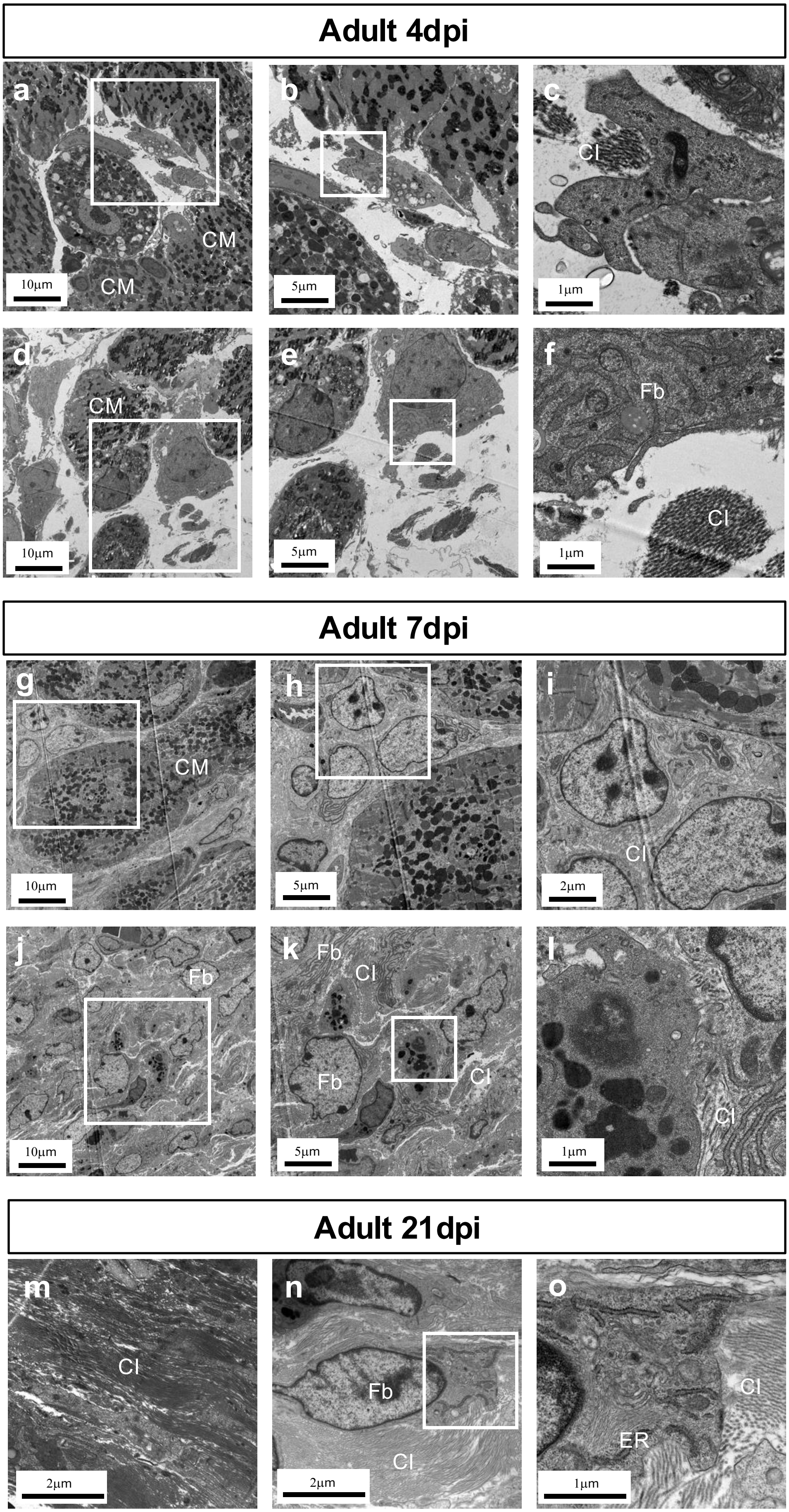

Supplementary Fig. 2. Transmission Electron microscopy of the ultrastructure of the infarct scar.

TEM showing cells and collagen fibrils in the adult post-MI hearts at 4dpi (a-f), 7dpi (g-l), and 21dpi (m-o). Dying cardiomyocytes and collagen fibrils were seen in lower magnification view of serial sections of the injured area within the myocardium of a heart at 4dpi (a, d), 7dpi (g, j) and 21 dpi (m). Higher magnified view from (a, d, g, j) showing different cell types and collagen fibrils (b, e, h, k). Higher magnified view from (b, e, h, k, n) showing ultrastructure of the cells and collagen fibril bundles (c, f, i, l, o). Scale bar: as indicated. CM: cardiomyocyte. Cl: collagen. Fb: fibroblast. ER: endoplasmic reticulum.

Supplementary Fig. 3. Periostin activation in P1 and P7 hearts post-MI.

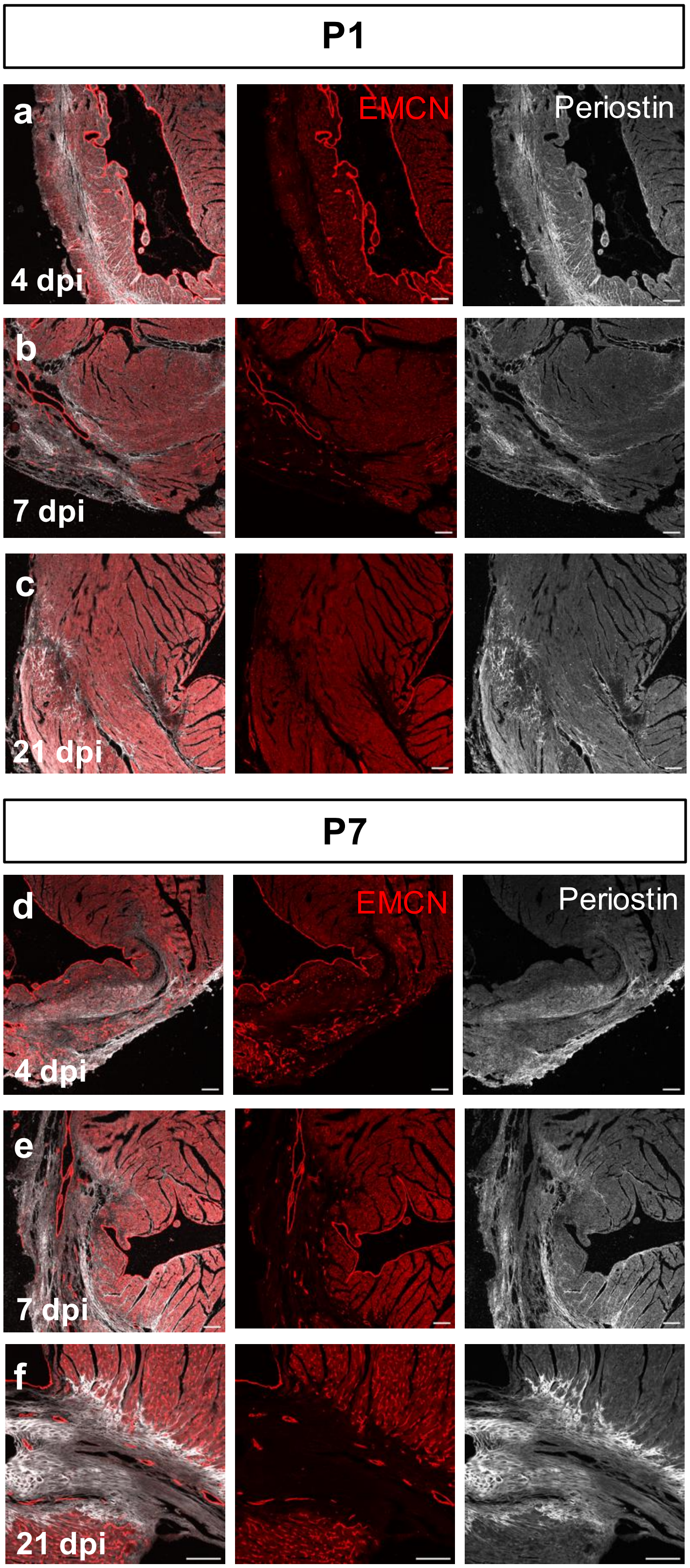

Supplementary Fig. 3. Periostin activation in P1 and P7 hearts post-MI.

Confocal imaging of immunofluorescent-stained serial sections from the P1 (a-c) and P7 (d-f) hearts showing expression of Periostin at 4dpi (a, d), 7dpi (b, e) and 21dpi (c, f). Periostin (white) is detected in the scar area demarcated by loss of vessel/endomucin-staining (EMCN, red). Sale bar: 100μm.

Supplementary Fig. 4. Periostin expression 1 day after injury in P1, P7 and adult hearts.

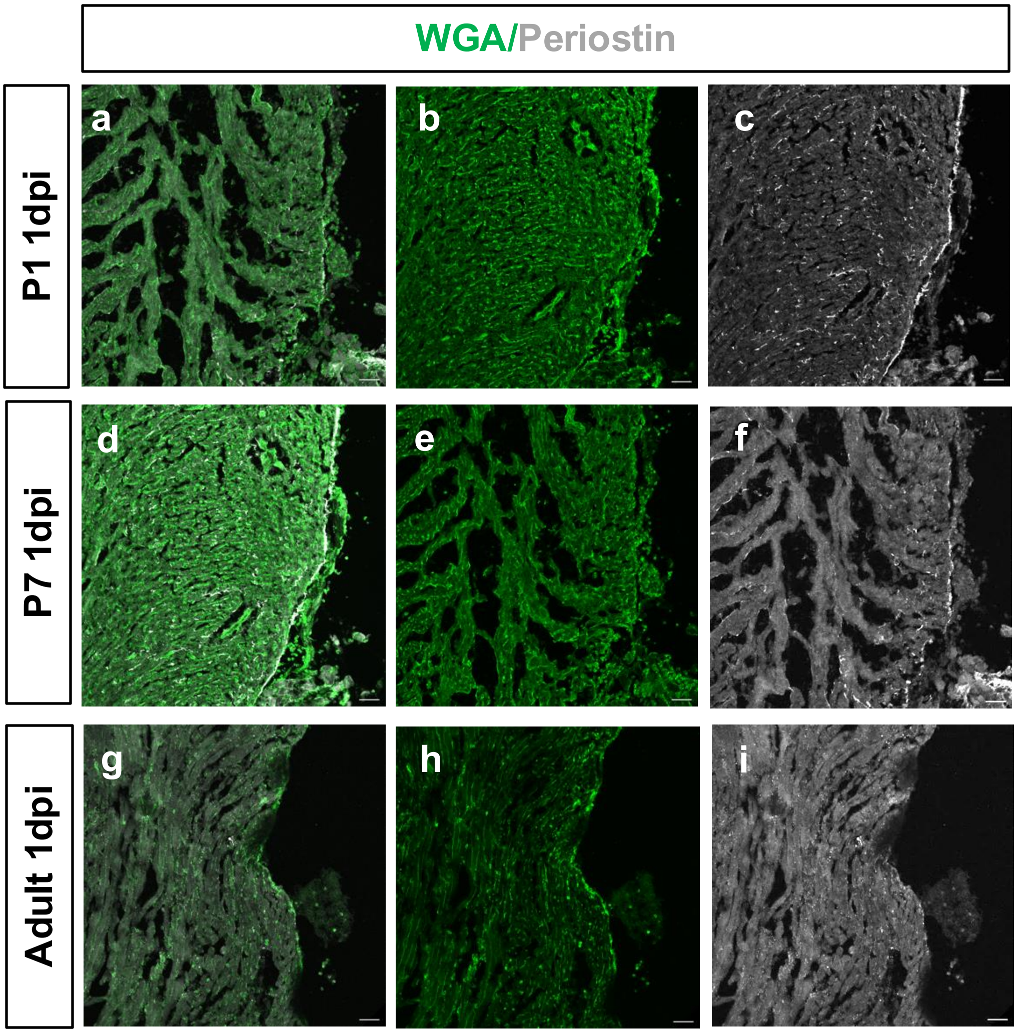

Supplementary Fig. 4. Periostin expression 1 day after injury in P1, P7 and adult hearts.

Confocal imaging of immunofluorescent-stained serial sections from the P1 (a-d), P7 (d-f) and adult (g-i). Fluorescent-conjugated WGA showed mild enrichment in the infarction area (green, (a, b, d, e, i). Periostin (white, g, f, i) was detected in epicardium proximal to infarction area and individual cells. Scale bar: 50 mm.

Supplementary Fig. 5. Single cell RNA-seq detected *Col5a1* in macrophage populations.

a

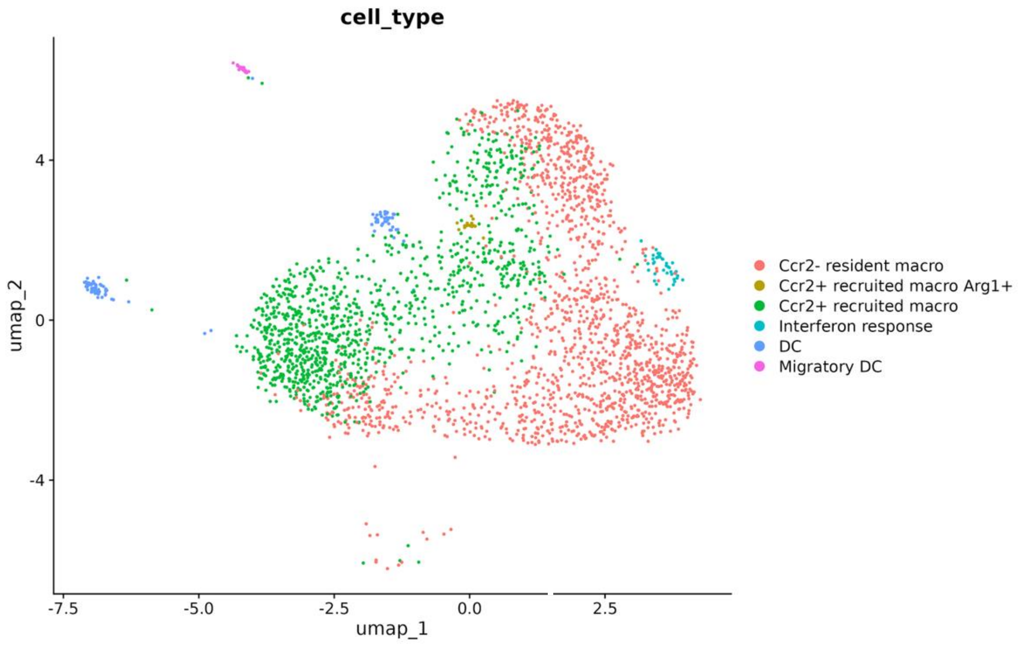

b

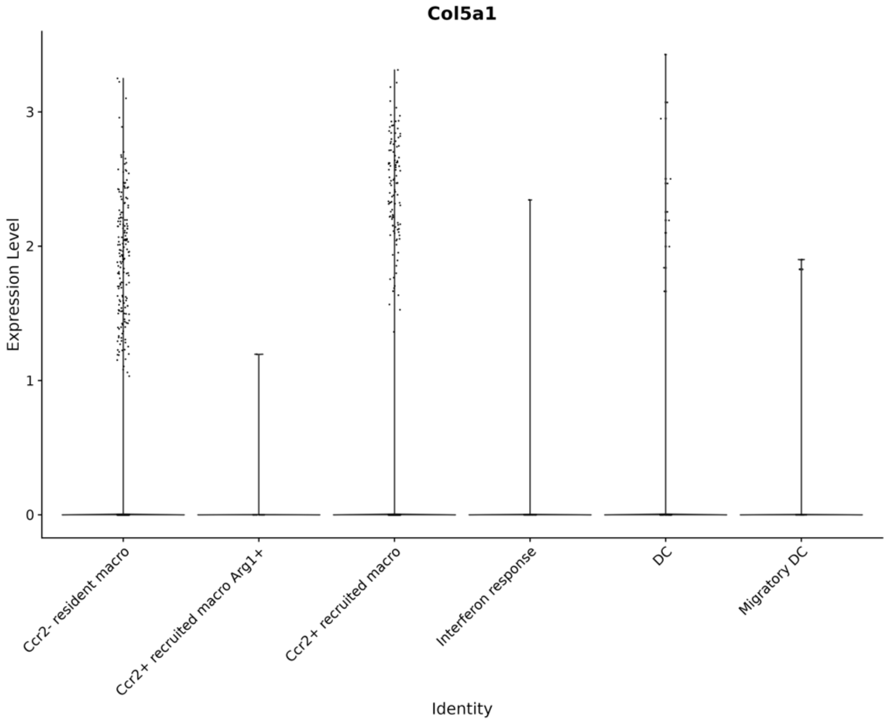

Supplementary Fig. 5. Single cell RNA-seq detected Col5a1 in macrophage populations.

Uniform manifold approximation and projection (UMAP) representation of macrophage and dendritic cell (DC) populations in mouse cardiac snRNA-seq data generated at 7 days post MI derived from the datasets generated by Ramadoss et al. (2024) (PMID: 39455836; a). The colour overlay distinguishes between tissue-resident cardiac macrophages (red) and recruited macrophages (brown and green) based on Ccr2 being mostly expressed in recruited macrophages. Violin plot showing expression of Col5a1 across macrophage populations b). Expression is evident in tissue resident cardiac and recruited macrophages.

Supplementary Fig. 6. hCD68-CreERT2 labelled tissue-resident macrophages 4-days after MI.

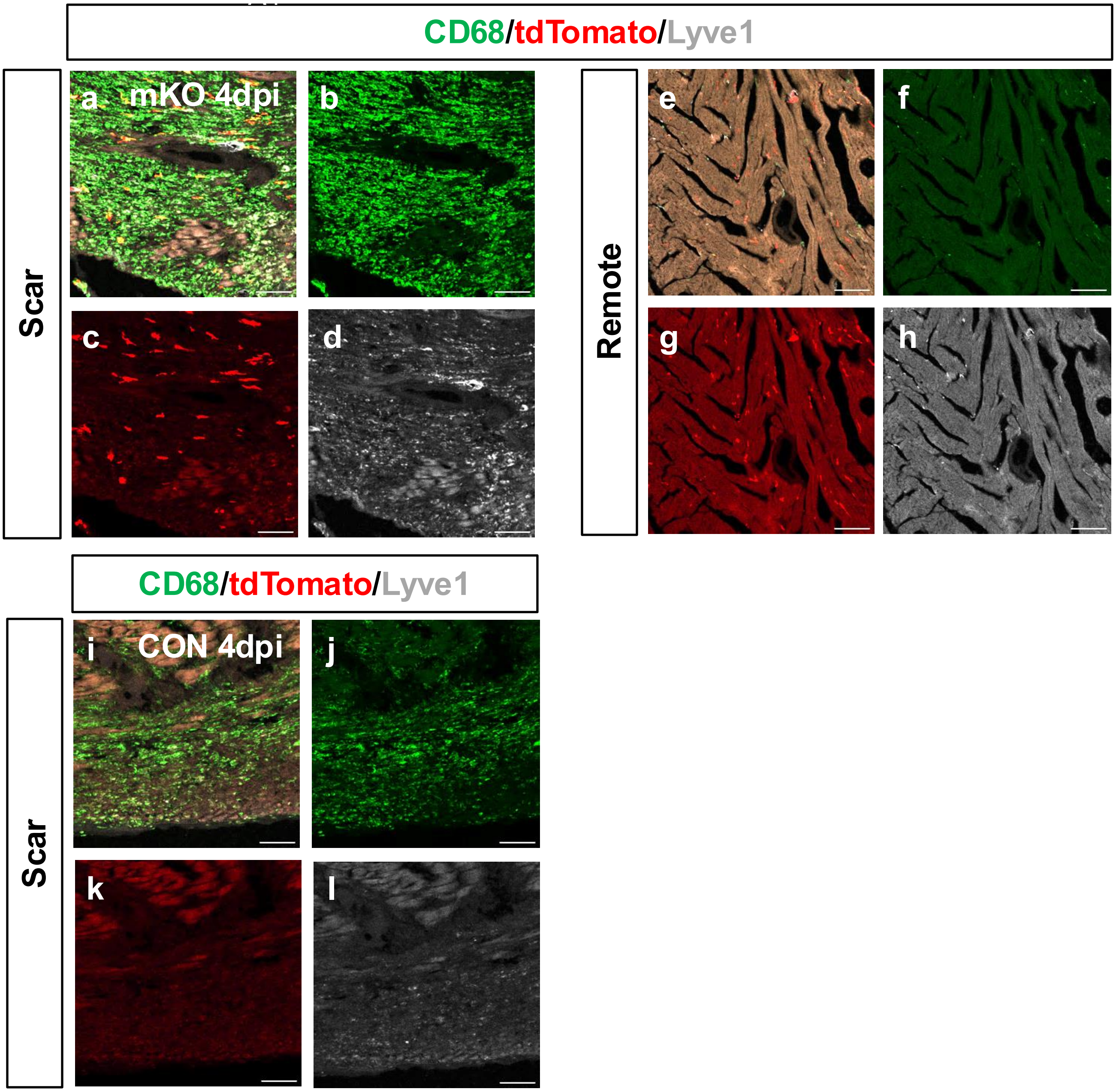

Supplementary Fig. 6. hCD68-CreERT2 labelled tissue-resident macrophages 4-days after MI.

Immunofluorescent staining of cross sections from a Col5a1mKO heart (a-h) and a control (CON) heart (i-l) at 4dpi, showing recruited macrophages (CD68, green), tdTomato positive cells (red) and resident macrophages (Lyve1, white) in the infarct region (a-d) and remote region in the left ventricle (e-h) as compared to the Cre negative control (i-l). . Scale bar: 100μm.

Supplementary Fig. 7. Col5a1 knock-down in CD68+ macrophages.

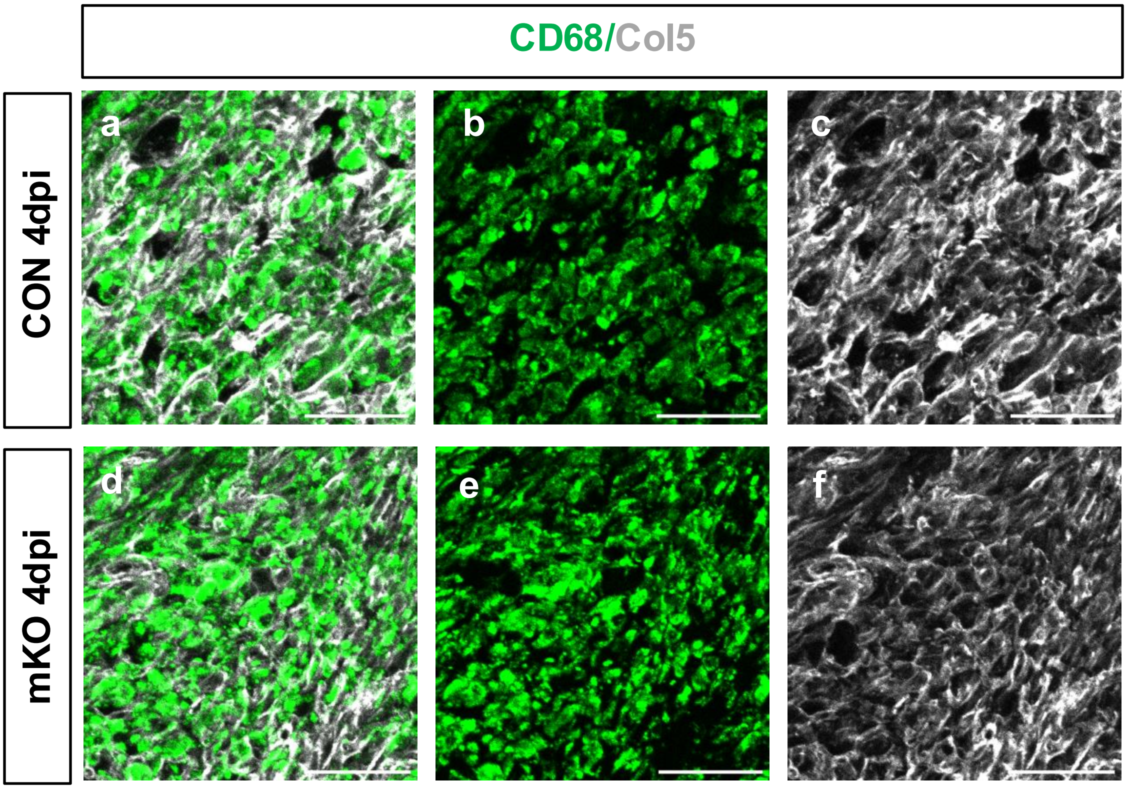

Supplementary Fig. 7. Col5a1 knock-down in CD68+ macrophages.

Immunofluorescent staining of cross sections from a control (CON) heart (a-c) and a Col5a1mKO (mKO) heart (d-f) at 4dpi, showing macrophages (CD68, green), and COL V (Col5, white) in the infarct region. Scale bar: 50μm.

Supplementary Fig. 8. hCD68-CreERT2 labelled macrophages 7-days after MI.

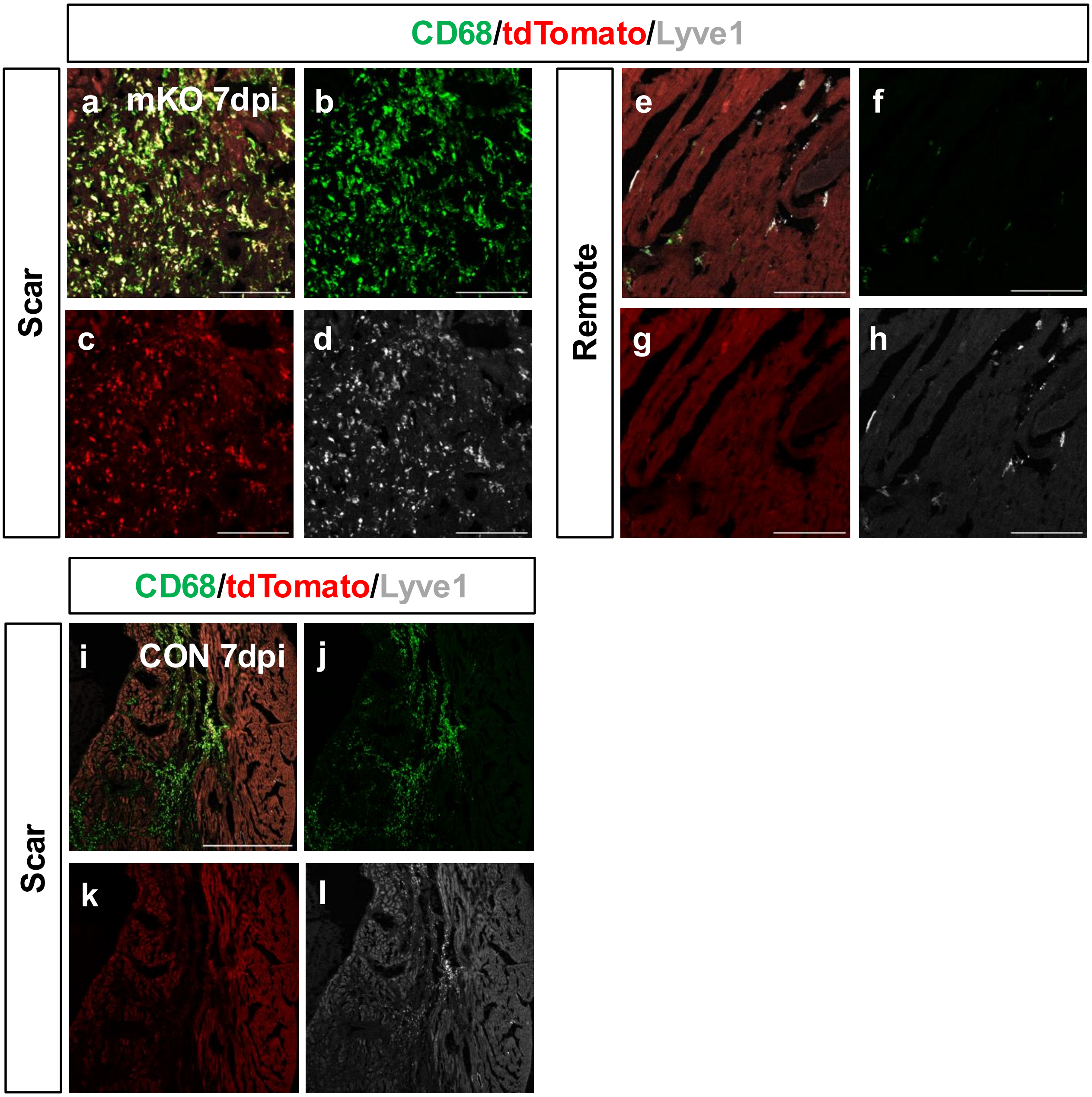

Supplementary Fig. 8. hCD68-CreERT2 labelled macrophages 7-days after MI.

Immunofluorescent staining of cross sections from a Col5a1mKO heart (a-h) and a control (CON) heart (i-l) at 7dpi, showing recruited macrophages (CD68, green), tdTomato positive cells (red) and resident macrophages (Lyve1, white) in the infarct region (a-d) and remote region (e-h) as compared to a Cre-negative control heart (i-l). Scale bar: 100μm.

Supplementary Fig.9. Col5a1 deletion did not compromise macrophage recruitment to the site of injury.

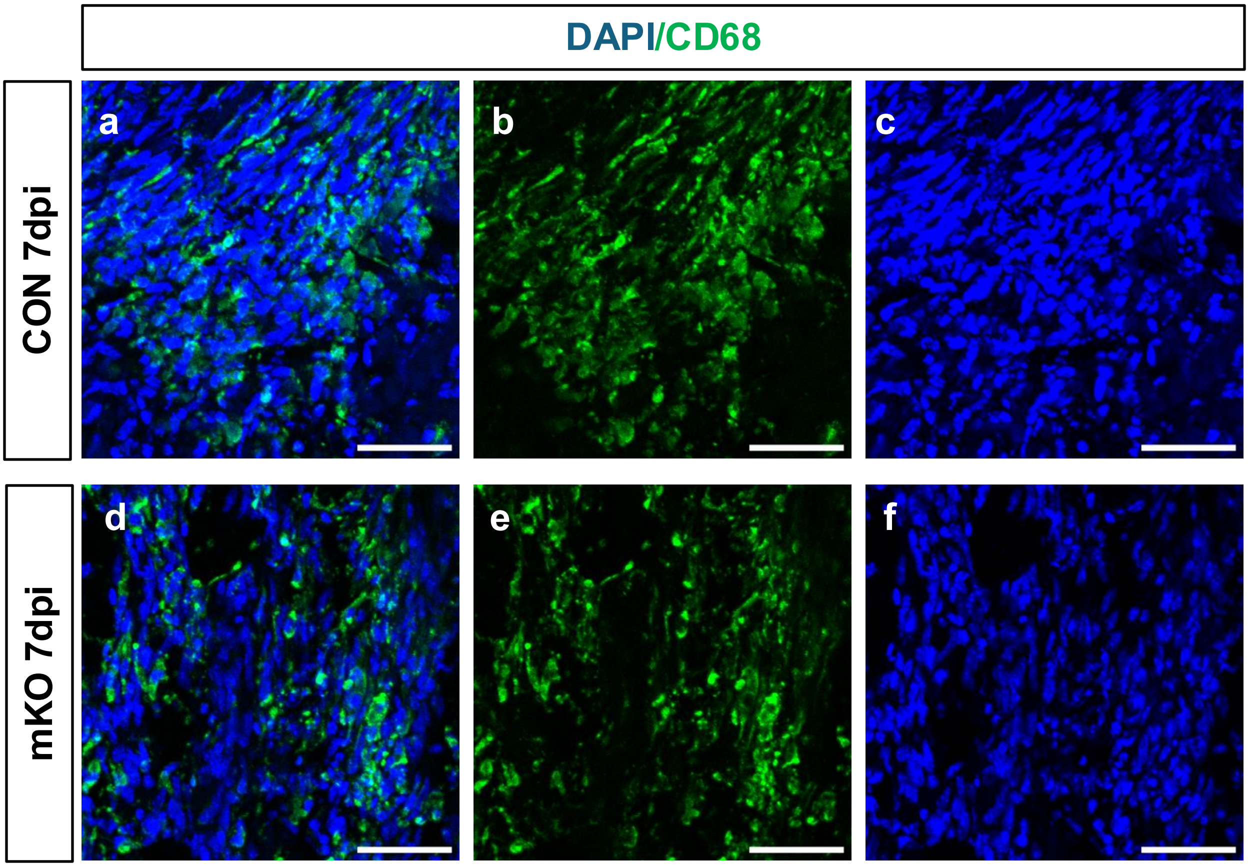

Supplementary Fig. 9. Col5a1 deletion did not compromise macrophage recruitment to the site of injury.

Immunofluorescent staining of cross sections from control (CON; a-c) and Col5a1mKO (mKO; d-f) hearts at 7dpi, showing macrophages (CD68, green), and DAPI (blue) in the infarct region. Scale bar: 100μm.

Supplementary Fig. 10. Expression of Periostin and Vimentin in Col5a1mKO mice.

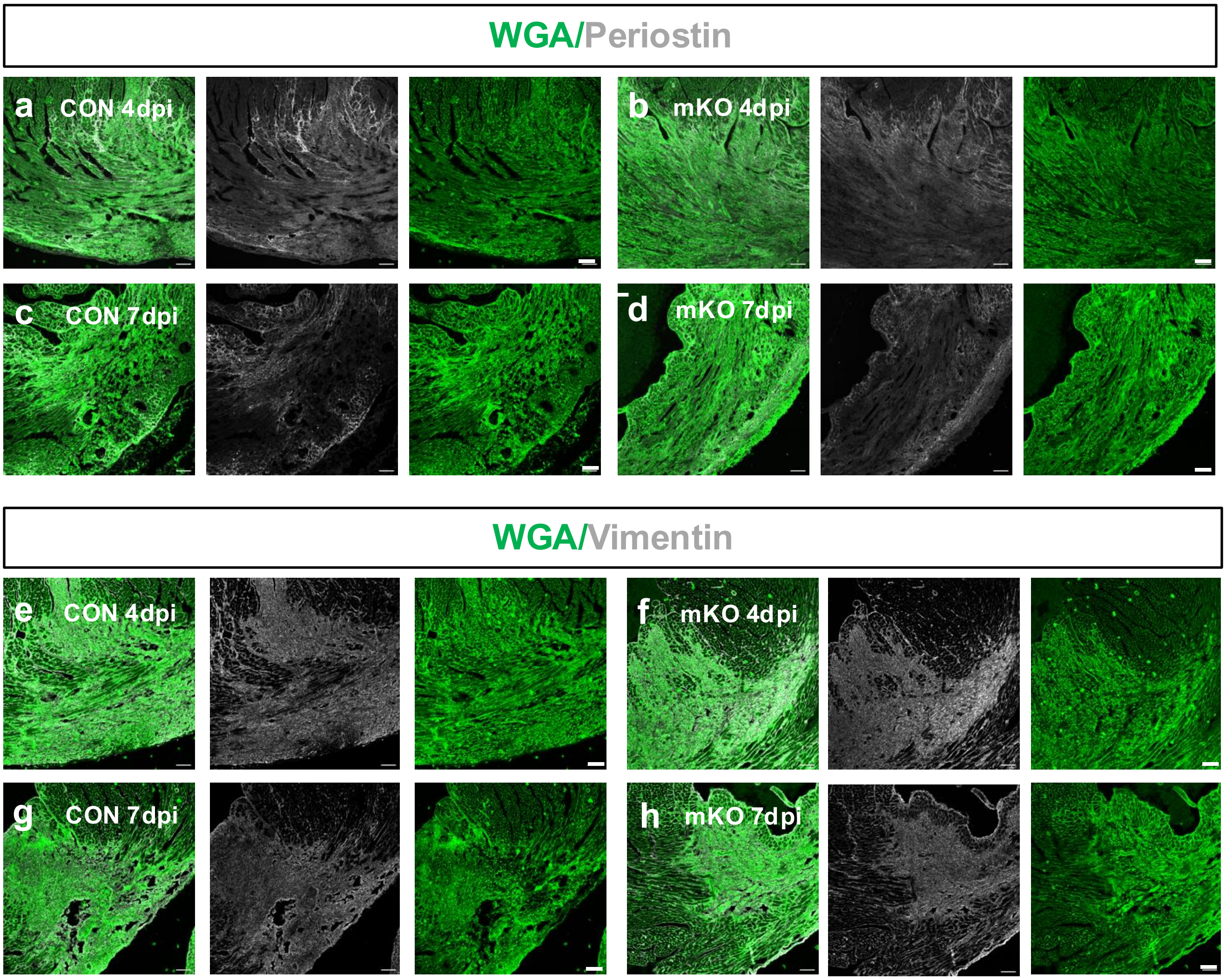

Supplementary Fig. 10. Expression of Periostin and Vimentin in Col5a1mKO mice.

Immunofluorescent staining of cross sections from a Col5a1mKO heart and a control (CON) showing expression of Periostin (a-d) and Vimentin (e-h) in the post-MI heart at 4dpi (a, b) and 7dpi (c, d). WGA (green) highlights the scar area. Periostin (white) and Vimentin (white) overlap with WGA. Representative images from n=3. Scale bar: 100μm.

Supplementary Fig. 11. Collagen deposition in Col5a1mKO hearts at different time points post-MI.

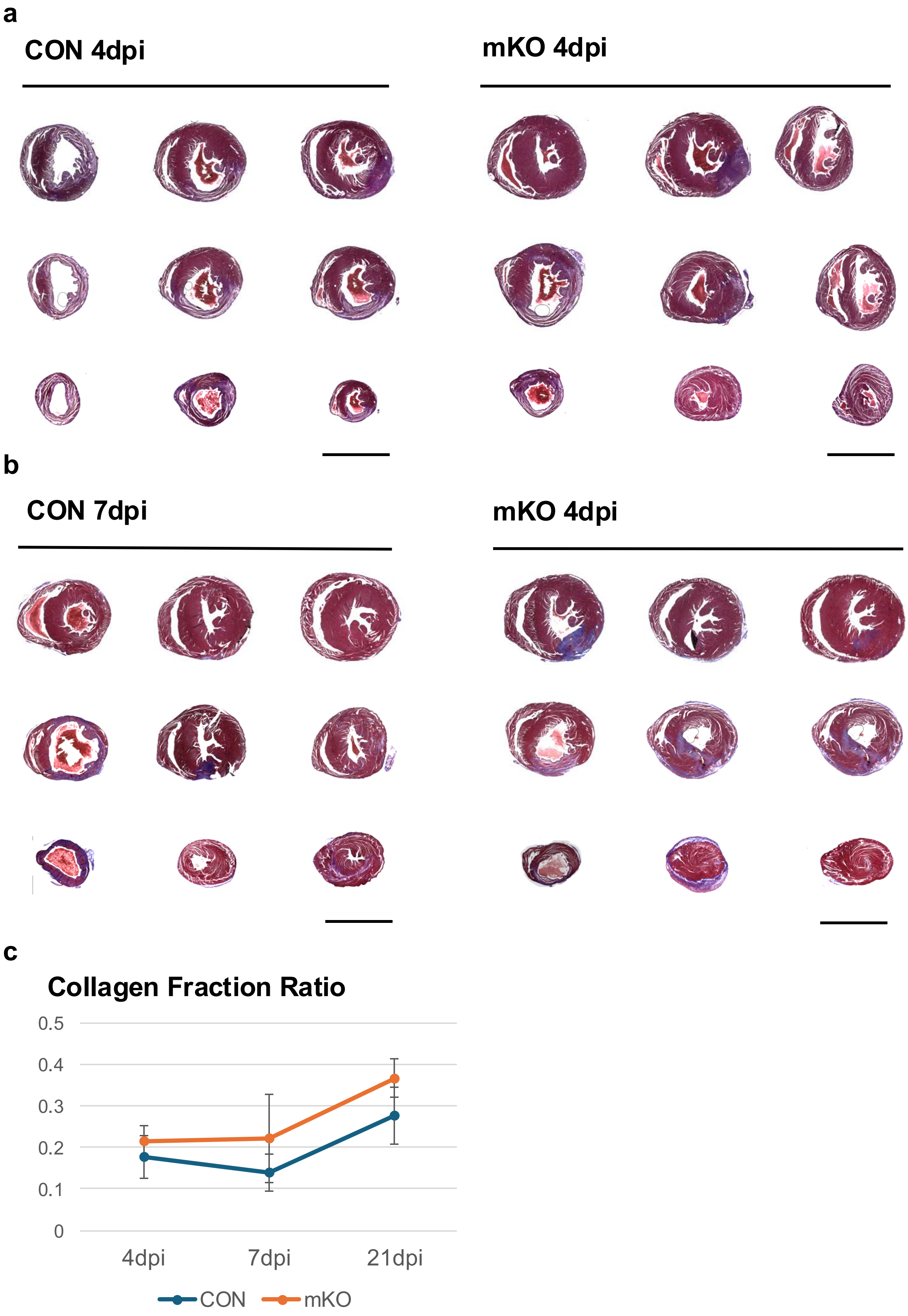

Supplementary Fig. 11. Collagen deposition in Col5a1mKO hearts at different time points post-MI.

Masson's Trichrome staining of serial sections of three Col5a1mKO post-MI hearts and their littermate controls at 4dpi (a) and 7dpi (b), respectively, to show differences in fibrosis and scarring. Scale bar: 5mm. Collagen fraction at different time points post-MI (c). N=3 per group; 4dpi, p=0.373; 7dpi, p=0.278; 21dpi, p=0.158.
